# High Performance Perovskite Solar Cells

**DOI:** 10.1002/advs.201500201

**Published:** 2015-12-02

**Authors:** Xin Tong, Feng Lin, Jiang Wu, Zhiming M. Wang

**Affiliations:** ^1^ Institute of Fundamental and Frontier SciencesUniversity of Electronic Science and Technology of ChinaChengdu610054P. R. China; ^2^ State Key Laboratory of Electronic Thin Films and Integrated DevicesUniversity of Electronic Science and Technology of ChinaChengdu610054P. R. China; ^3^ Department of Electronic and Electrical EngineeringUniversity College LondonTorrington PlaceLondonWC1E 7JEUnited Kingdom

**Keywords:** electron‐transporting materials, hole‐transporting materials, perovskite solar cells, photovoltaic parameters

## Abstract

Perovskite solar cells fabricated from organometal halide light harvesters have captured significant attention due to their tremendously low device costs as well as unprecedented rapid progress on power conversion efficiency (PCE). A certified PCE of 20.1% was achieved in late 2014 following the first study of long‐term stable all‐solid‐state perovskite solar cell with a PCE of 9.7% in 2012, showing their promising potential towards future cost‐effective and high performance solar cells. Here, notable achievements of primary device configuration involving perovskite layer, hole‐transporting materials (HTMs) and electron‐transporting materials (ETMs) are reviewed. Numerous strategies for enhancing photovoltaic parameters of perovskite solar cells, including morphology and crystallization control of perovskite layer, HTMs design and ETMs modifications are discussed in detail. In addition, perovskite solar cells outside of HTMs and ETMs are mentioned as well, providing guidelines for further simplification of device processing and hence cost reduction.

## 1 Introduction

**Figure 1:**
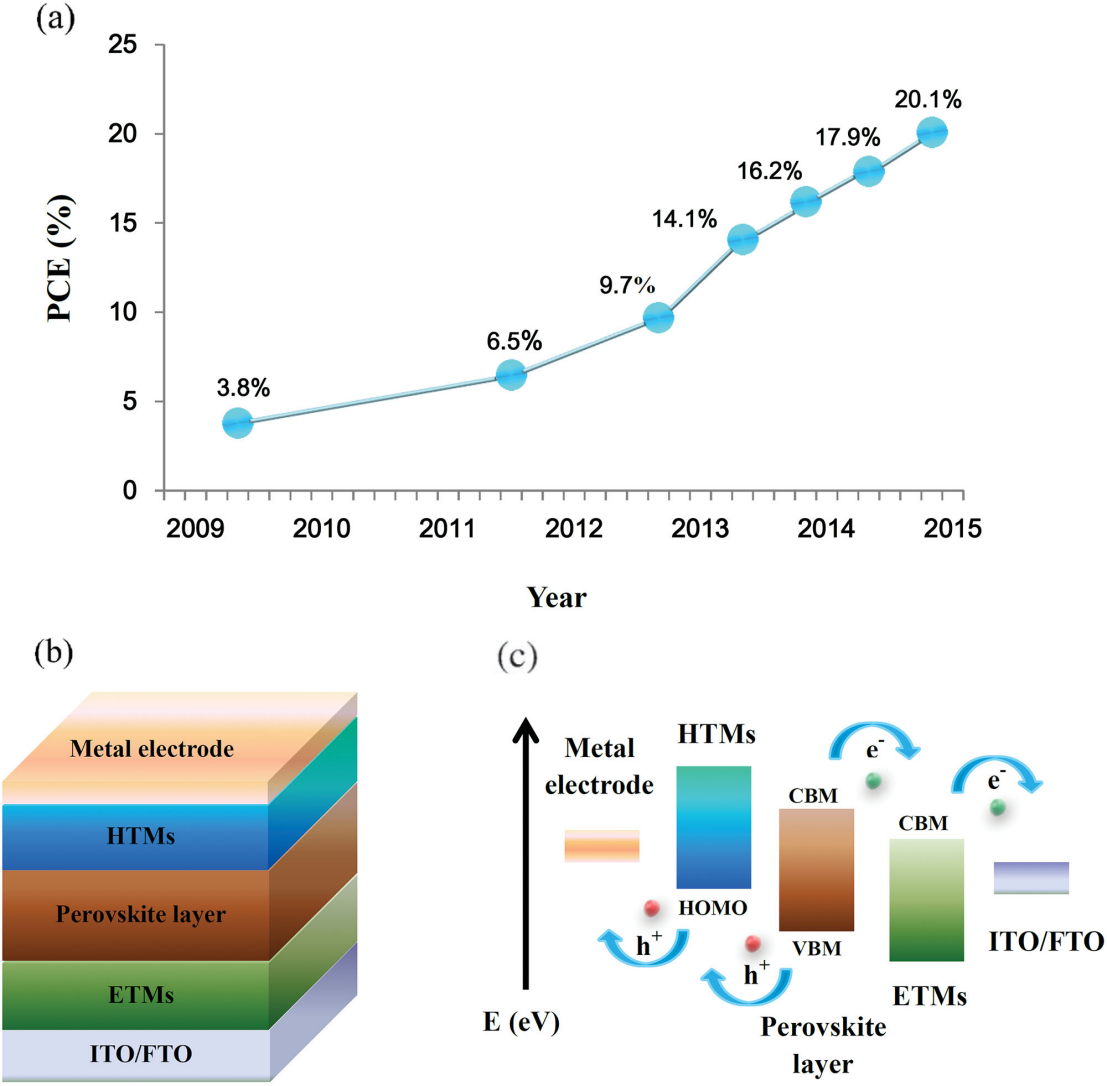
a) Rapid PCE evolution of perovskite solar cells from 2009 to 2015. b) Schematic diagram of general device configuration. c) Work principle of normal perovskite solar cells based on (b).

The third generation of solar cells, such as dye‐sensitized solar cells, quantum dot solar cells and organic solar cells, have been intensively explored aiming towards lowering device costs and enhancing power conversion efficiency (PCE; measured under one sun illumination unless supplementarily stated in this paper).^1^ Nevertheless, the PCEs of these solar cells are mostly poor (≈10%–12%) to date, primarily owing to imperfect device fabrication, low light absorbing efficiency and unexpected charge recombination.^2^ Holding promise for addressing these problems, novel perovskite materials based on organometal halide CH3NH3PbX3 (CH3NH3^+^ = MA, X = I, Cl and Br)^3^ are developed and their suitable attributes for solar cells including strong light absorption, bipolar transporting abilities, balanced electron and hole diffusion lengths, lower charge recombination and facile fabrication are demonstrated as well.^4^ Solar cells utilizing these perovskite materials (i.e., perovskite solar cells) have emerged as a rising star since being first investigated by Miyasaka and his colleagues in 2009, who employed perovskite material as a sensitizer in liquid‐dye‐sensitized solar cells and demonstrated a relatively low PCE of around 3.8%.^5^ Afterwards, a stable and all‐solid‐state mesoscopic device with a PCE of 9.7% was fabricated in 2012.^6^ In 2013, Julian et al. used sequential deposition method to attain optimized morphology of perovskite layer and the PCE of the device reached up to 14.1%.^7^ Late in the same year, a PCE of 16.2% was achieved by applying hybrid halogen elements of perovskite layer.^8^ Recently, a record PCE of 20.1% was reported by Korean Research Institute of Chemical Technology (KRICT),^9^ which has already been certified by National Renewable Energy Laboratory (NREL) in late 2014,^10^ showing the remarkably rapid developments of PCE over just five years (illustrated in **Figure** **1**a).^11^

General configuration of perovskite solar cells is depicted in Figure 1b, that is, perovskite layer is sandwiched between a hole‐transporting material (HTM) and an electron‐transporting material (ETM), along with additional metal electrode and tin‐doped indium oxide (ITO)/fluorine‐doped tin oxide (FTO) substrates. Two main device architectures are mesoscopic structure utilizing mesoporous metal oxide materials (TiO_2_ et.al) and planar heterojunction structure without mesoporous materials.^12^, ^13^, ^14^ As described in Figure 1c, these perovskite solar cells work efficiently according to following processes: perovskite layer absorbs the incident light, generating electron and hole, which are extracted and transported by ETMs and HTMs, respectively. These charge carriers are finally collected by electrodes forming perovskite solar cells. Particularly, in order to realize efficient hole and electron transport and extraction, the highest occupied molecular orbital (HOMO) of HTMs should be higher than valence band maximum (VBM) of perovskite materials and the conduction band minimum (CBM) of ETMs should be lower than CBM of perovskite materials.^15^

The perovskite layer, which acts as a light absorber, is expected to possess efficient surface coverage and large grain size to obtain high performance perovskite solar cells. Therefore, the key points of techniques employed for preparing perovskite layers (such as conventional spin‐coating and vapor deposition) are how to achieve better morphology while simultaneously being facile, cost‐effective and environment friendly. Composition tuning of perovskite materials is feasible as well, strongly affecting quality of as‐prepared perovskite layer, which in turn impacts the consequent device performance. HTMs, which are adjacent to perovskite layer, play a very important role in hole collection and transportation. Therefore, various organic HTMs are studied by molecular engineering and design facing the challenges for dopant‐free, high charge carrier mobility and stable attributes. Inorganic HTMs are also investigated towards appropriate band gap and doping strategy. Except for HTMs, novel nanostructured ETMs are explored and modifications like interface engineering are essential regarding enhanced electron extraction and transportation. Moreover, HTM‐free and ETM‐free architectures are promising configurations, but still necessitate optimizations for future low‐cost perovskite solar cells with respect to the relatively low PCE of as‐fabricated perovskite solar cells.

In this review, notable progress is described according to the three main parts of perovskite solar cells: perovskite layer, HTMs and ETMs. HTM‐free and ETM‐free configurations are also mentioned, which holds prospects for future device simplification and cost reduction. Challenges and strategies for achieving high PCE and enhancing other photovoltaic parameters of perovskite solar cells are discussed, providing guidelines for prospective device optimization. In addition, future improvements of perovskite solar cells are proposed.

## 2 Perovskite Layer

Controllable morphology and crystallization of perovskite layer (light harvester) have received enormous attention due to their intimate effects on performance of perovskite solar cells. For morphology, inhomogeneous perovskite layer leads to pin‐hole formation and incomplete surface coverage, resulting in increased low‐resistance shunting paths and inefficient light absorption in devices. As for crystallization, improved crystalline quality of perovskite layer gives rise to reduced charge carrier trapping and defect density, which is favorable for solar cell performance.^16^, ^17^ Therefore, strategies are concentrating on evolution of preparation methods for large grain size, pin‐hole free and uniform perovskite layer, which potentially results in significant light absorption, charge carriers generation, and further improved optoelectronic properties of devices. For example, homogeneous perovskite layer with sufficient surface coverage has been demonstrated to enhance efficiency of perovskite solar cells. In this work, morphology of perovskite layer can be controlled via applying various growth parameters during solution‐processed technique. It is found that devices realized the highest photocurrent and PCE only with the best surface coverage of perovskite layer due to its optimized morphology.^18^

Following investigations are proposed regarding controllable perovskite layer preparation. Xiao et.al introduced chlorobenzene as second solvent to prompt a fast‐crystallization process during perovskite MAPbI_3_ spin‐coating, which is demonstrated to be a facile method to prepare highly uniform MAPbI_3_ layer. By means of such facilitated crystallization, the grain size of MAPbI_3_ reached the magnitude of micrometers and as‐fabricated perovskite solar cells obtained a PCE of 14% in average.^19^ Zhao et.al utilized thermal decomposition of film formed by PbI_2_, MABr, and MACl to prepare uniform MAPbI_2_Br‐based perovskite layer. The thermal decomposition process was found to strongly affect the final film morphology, further influencing device performance.^20^ Xie and co‐workers have demonstrated that vacuum thermal annealing is another promising method to control perovskite layer crystallization. Herein, MACl is suggested to be important in film preparation and stabilization of device. Careful control of MACl release is feasible by vacuum annealing at appropriate temperature, resulting in pinhole‐free perovskite layer with no chloride‐based residues. Related perovskite solar cell fabricated with as‐treated layer exhibits a PCE as high as 13.6% and improved stability.^21^ Additionally, annealing atmosphere plays an important role in crystallization. For instance, annealing perovskite layer in air results in optimal grain size and morphology as compared to annealing in nitrogen, which is mainly caused by the electronic disorder.^22^ It is verified that perovskite layer annealed in nitrogen could attain smaller grain size with higher degree of electronic disorder as compared to annealed in air. This higher degree of electronic disorder gives rise to shorter photoluminescence lifetimes and lower photoluminescence quantum efficiency, which are undesired in perovskite solar cells, thus resulting in degradation of device performance.

Huang's group have investigated perovskite layer prepared by interdiffusion of MAI and PbI_2_.^23^ Note that it is a simple, low temperature approach, which forms uniform and porous‐free perovskite layer and as‐prepared MAPbI_3_ layer showed a high PCE of 15.4% when utilized in perovskite solar cell. Furthermore, they studied annealing of MAPbI_3_ perovskite layer with N, N‐dimethylmethanamide (DMF) based on interdiffusion process and claimed that annealing was also a facile and effective method to control the crystallization and grain size of perovskite layer MAPbI_3_. The improvement of electronic properties after annealing led to a perovskite solar cell with a high PCE of 15.6%, which is mainly attributed to the enhanced charge diffusion length from several hundred nanometers to over 1 μm.^24^

**Figure 2:**
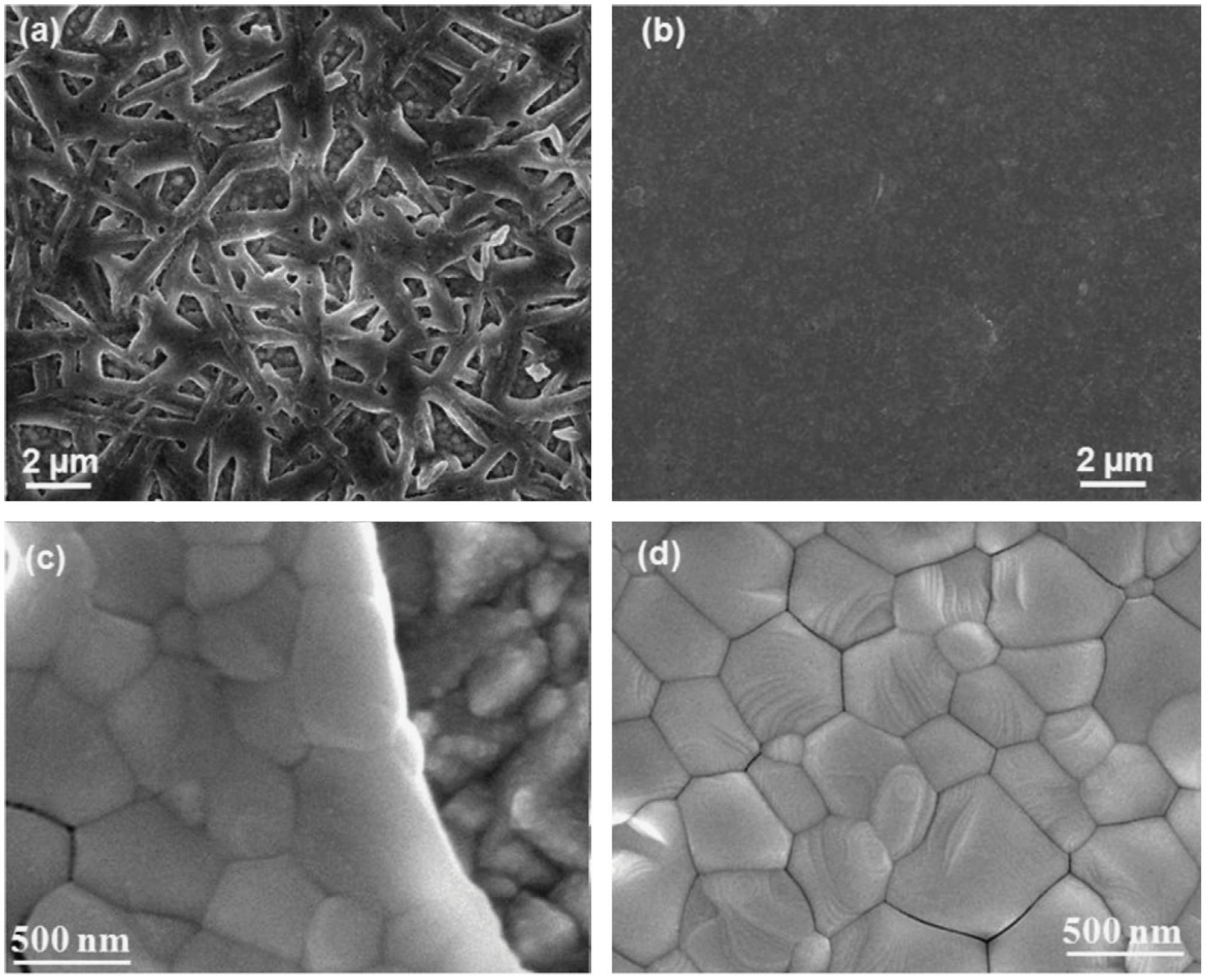
SEM images of perovskite layers prepared by a,c) traditional spin‐coating technique and b,d) gas‐assisted technique . Images (c) and (d) are high magnification images with respect to (a) and (b). Reproduced with permission.^26^ Copyright 2014, Elsevier.

In addition, gas‐assisted techniques are proved to be an efficient method for high quality perovskite layer formation. In an investigation by Chen et al., organic MAI gas flow is used to interact with PbI2 layer and form homogeneous and large grain sized MAPbI_3_ layer, thus enabling high performance (PCE ≈ 12%) perovskite solar cell.^25^ Another unique gas‐assisted method, which introduces argon gas flow during spin‐coating of MAPbI_3_, is also demonstrated to produce highly dense and uniform perovskite layer containing grains with decent quality in comparison with traditional spin‐coating perovskite layer, which is depicted in **Figure** **2**. Figure 2a and Figure 2c are scanning electron microscope (SEM) images of perovskite layers prepared by spin‐coating technique. In contrast, SEM images (Figure 2b and Figure 2d) of perovskite layers prepared by gas‐assisted technique exhibit more homogeneous and compact surface morphology, thus resulting in faster charge carrier transport and an average PCE reaching 16% in subsequent perovskite solar cell.^26^ In addition, Zhu et al. claimed that DMF vapor‐assisted technique (fumigation) is a novel and simple method to prompt self‐repair recrystallization of perovskite layer and obtain high quality film for high performance devices.^27^ Gas‐assisted crystallization has also been demonstrated to form optimal surface morphology as compared to direct solution process in perovskite solar cell with identical configuration,^28^ manifesting the importance of gas‐assisted technique in perovskite layer preparation.

In the previous work of Liu et al., perovskite layer can be prepared by a dual source vapor deposition system and shows a superb PCE over 15% in related perovskite solar cell.^29^ However, high cost in vacuum system hampers its commercial application. To find a low‐cost method, low‐pressure chemical vapor deposition (LPCVD) has been utilized to successfully prepare homogeneous perovskite layer without expensive vacuum facilities. In this LPCVD process, CH_3_NH_3_I powder was placed in quartz boat as vapor source and heated to react with PbI_2_ films, which was also placed in furnace. With a controllable reaction rate, the as‐prepared perovskite layer possesses uniform morphology, high crystalline quality and unique moisture‐resistant property, producing a perovskite solar cell with a PCE of 12.8%. Such LPCVD method facilitates further investigation on facile and low‐cost thermal vapor deposition of efficient perovskite solar cells.^30^

Sequential spin‐coating has already been investigated as an efficient approach in perovskite solar cell fabrication, rendering outstanding photovoltaic performance.^7^ To this end, J. H. Im and his colleagues prepared perovskite MAPbI_3_ cuboids by sequential spin‐coating and studied influences of different cuboid sizes on as‐fabricated perovskite solar cells. It is found that the MAI concentration was the main parameter to affect the MAPbI_3_ cuboid size and device performance, which leads to a high PCE up to 16% in average.^31^

**Figure 3:**
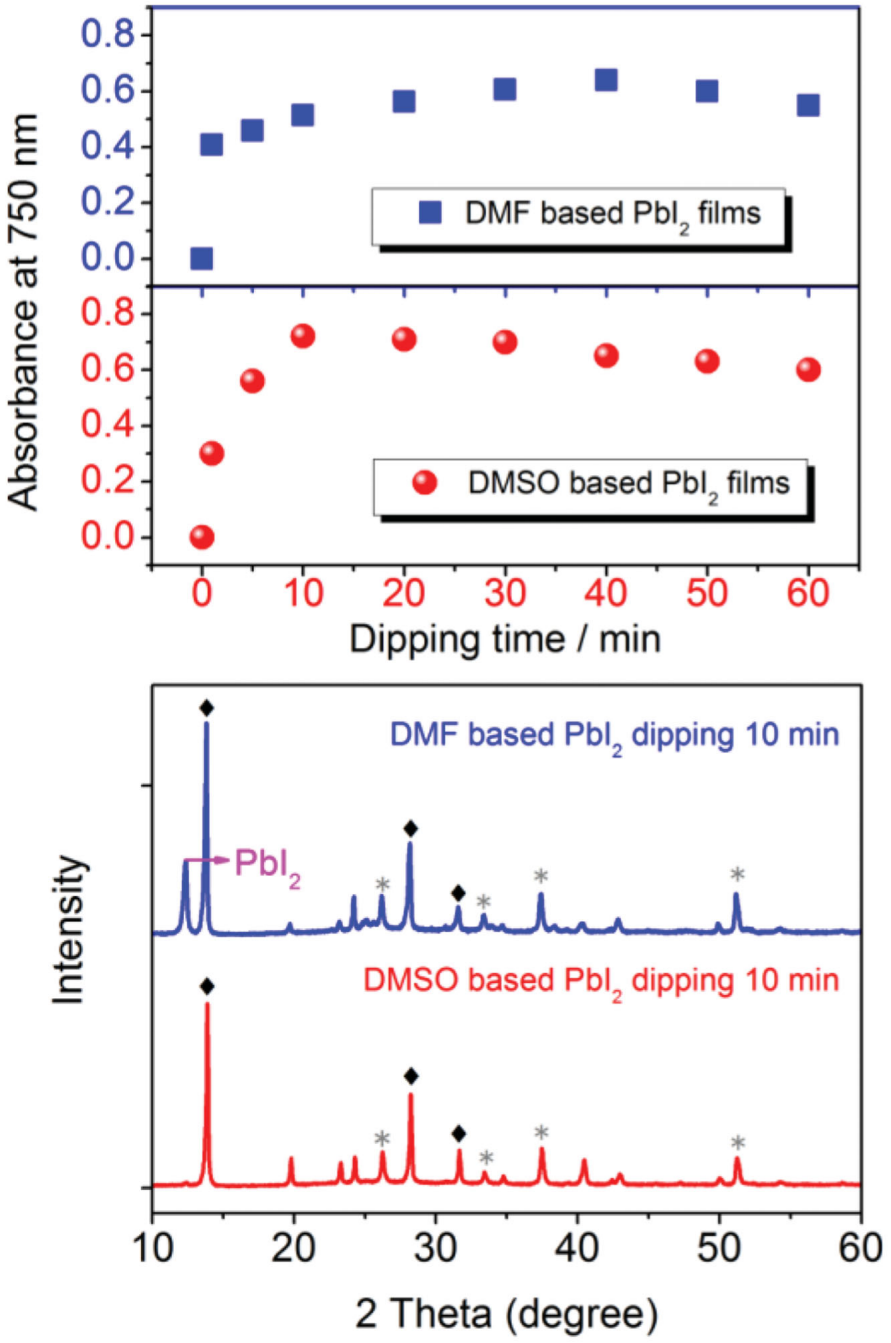
a) Variation of layer absorbance at 750 nm as a function of dipping time corresponding to conventional DMF–PbI_2_‐based (blue) and as‐prepared DMSO–PbI_2_ ‐based perovskite layer (red). b) X‐ray diffraction (XRD) pattern of DMF–PbI*2*‐based and DMSO–PbI_2_‐based perovskite layer after 10 min dipping. Reproduced with permission.^33^ Copyright 2014, Royal Society of Chemistry.

To avoid incomplete conversion of precursor PbI2 in sequential deposition and effectively control the grain size of as‐deposited MAPbI_3_ layer,^7^ a gas‐solid crystallization of the MAPbI_3_ layer is employed to reduce its interaction with additives and water, which forms uniform layer and hinders charge recombination.^32^ Also, solvent engineering of solution‐processed perovskite layer is proved to address problems of sequential deposition and achieve highly efficient perovskite solar cells. In this regard, Wu et al. employed dimethylsulfoxide (DMSO) as solvent to dissolve PbI_2_, which exhibits stronger interaction with PbI_2_ compared with conventionally used DMF, thus hindering the crystallization of PbI_2_ and giving rise to formation of amorphous perovskite layer. The as‐formed amorphous PbI_2_ can be deposited with MAI to form homogeneous MAPbI_3_ layer and correlated process is illustrated in **Figure** **3**. Figure 3a depicts the absorbance at 750 nm (represent amount of formed perovskites) as a function of dipping time, showing that even though DMF‐PbI_2_ layer reacted with MAI solution in a shorter time initially, the eventual results were almost consistent with respect to DMSO–PbI_2_ layer reacting with MAI solution. After 10 min dipping of two different precursors to form perovskite layer, X‐ray diffraction (XRD) patterns were measured and shown in Figure 3b, which denoted the extra peak and elucidated the residue of PbI_2_ in conventional DMF–PbI_2_‐based perovskite layer as compared to DMSO–PbI_2_‐based perovskite layer, thus demonstrating the incomplete conversion of PbI_2_ and highlighting the improved crystallization with usage of DMSO.^33^ Besides, Jeon et al. utilized mixture of γ‐butyrolactone (GBL) and DMSO with toluene drip during spin‐coating to prepare highly uniform and compact MAPb(I_1–*x*_Br*_x_*)_3_ perovskite layer,which is ascribed to the function of MAI–PbI_2_–DMSO intermediate phase, thus attaining a high performance perovskite solar cell with a notable PCE of 16.2%.^8^

**Figure 4:**
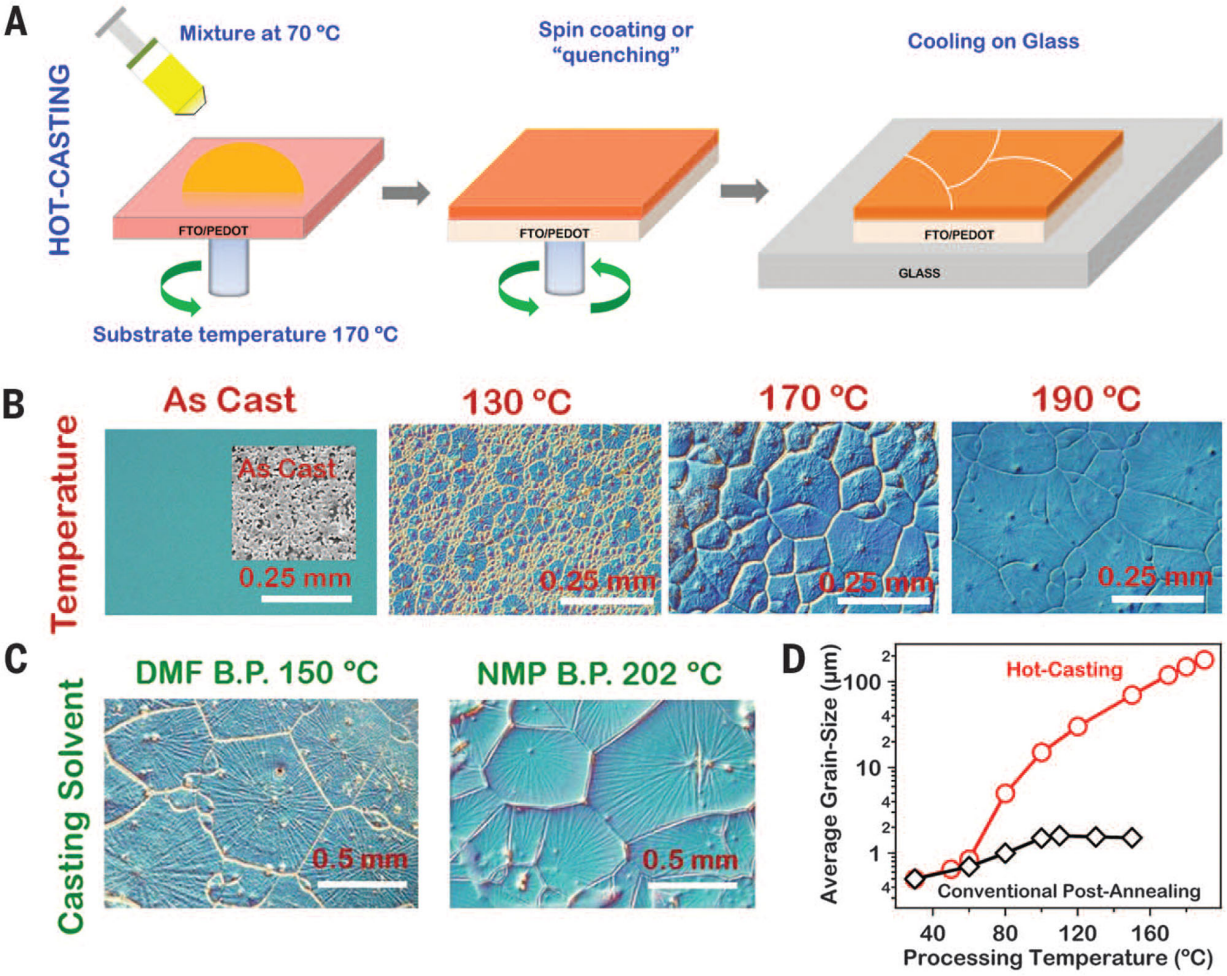
a) Schematic diagram of hot‐casting process. b) Optical images (resolution: 0.25 mm) of as‐casted perovskite layer under different substrate temperature (130 °C, 170 °C and 190 °C). c) Optical images of films with additives of DMF (boiling point: 150 °C) and NMP (boiling point: 202 °C). d) Average grain size as a function of processing temperature by hot‐casting method (red curve) and traditionally annealing method (black curve). Reproduced with permission.^34^ Copyright 2015, American Association for the Advancement of Science.

Nie et al. employed a solution based hot‐casting technique and achieved large‐scale grains of perovskite layer in the order of magnitude of millimeter, as exhibited in **Figure** **4**. The hot‐casting process is shown in Figure 4a; PbI_2_ and MACl solution at 70 °C was casted onto the poly(3,4‐ethylenedioxythiophene) polystyrene sulfonate (PEDOT:PSS)/FTO substrate (170°C) and spin‐coated for 15 s. After cooling down on glass, homogeneous and large‐scale perovskite layer (MAPbI_3–*x*_Cl*_x_*) was formed. To observe the grain morphology during 70 °C solution‐based processing, Figure 4b exhibits optical images (0.25 mm resolution) of as‐casted perovskite layer under different substrate temperature (130 °C, 170 °C and 190 °C) and Figure 4c illustrates two different optical images with additives of DMF (boiling point: 150 °C) and N‐methyl‐2‐pyrrolidone (NMP) (boiling point: 202 °C). In addition, average grain size as a function of processing temperature in this hot‐casting method and traditional annealing method are shown in Figure 4d. It is demonstrated that grain size of the as‐prepared perovskite layer is larger and reaches the order of magnitude of millimeter with increasing substrate temperature along with incorporation of DMF and NMP, which results in a very high PCE of 18% after device fabrication.^34^ Notably, this work is consistent with the study by Xu et al. that MACl plays a critical role in enhancing crystallization quality of perovskite layer.^12^

In order to investigate the composition of perovskite layer for better photovoltaic performance, Zhang et al. altered the anions of precursor PbI_3_ and PbCl_3_ by actinium. It is found that PbAc_3_ without halogen could help control the crystallization process, which benefits crystal growth dynamics and obtains uniform perovskite film with better surface coverage, leading to a future topic in exploring other elements to replace halogen and produce higher performance perovskite solar cells.^35^

**Figure 5:**
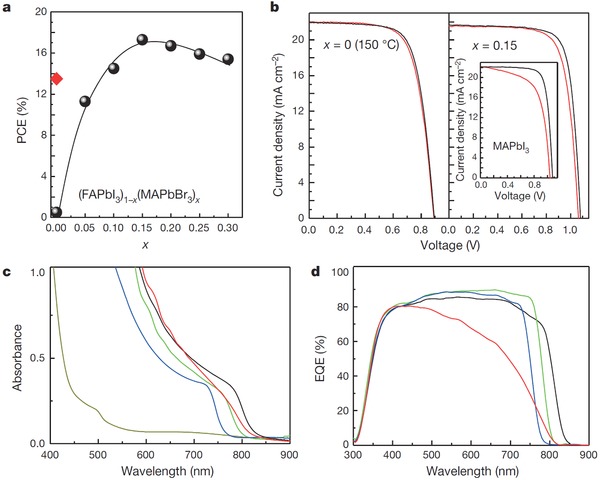
a) The PCE as a function of *x* (varied from 0 to 0.3) in (FAPbI3)_1–*x*_ (MAPbBr_3_)*_x_*‐based solar cells (100 °C annealing); the red point is the PCE of initial solar cell only with perovskite FAPbI_3_ (150 °C annealing). b) *J–V* curves of (FAPbI_3_)_1–*x*_ (MAPbBr_3_)*_x_*‐based devices with *x* = 0 (150 °C annealing), 0.15 and referential MAPbI_3_ device. c) UV–vis absorption spectra corresponding to *x* = 0 (dark yellow curve), 0.05 (red curve), 0.15 (green curve) and 0.25 (blue curve); black curve represents FAPbI_3_ (150 °C annealing) and d) correlated EQE spectra. Reproduced with permission.^37^ Copyright 2015, Nature Publishing Group.

Replacing MA composition of MAPbI_3_ with formamidinium (HN = CHNH_3_^+^, FA) to form perovskite (MA)*_x_*(FA)_1–*x*_PbI_3_ (*x* = 0–1) has been studied by Pellet et al., (MA)*_x_*(FA)_1–*x*_PbI_3_ has been proved to possess better photovoltaic performance than MAPbI_3_, which is attributed to the red‐shifted absorption spectrum inducing extra light harvesting.^36^ Following this investigation, (FAPbI_3_)_1–*x*_(MAPbBr_3_)*_x_*–based solar cell has been studied by Seok et al.. The solar cell results are exhibited in **Figure** **5**. Figure 5a illustrates the PCE as a function of *x* (varied from 0 to 0.3) in (FAPbI_3_)_1–*x*_(MAPbBr_3_)x–based solar cells (100 °C annealing for 10 min), showing a maximum PCE value at *x* = 0.15. The red point is the PCE of initial solar cell only with perovskite FAPbI_3_ (150 °C annealing for 10 min). Current density–voltage (*J–V*) curves of devices with *x* = 0 (150 °C annealing), 0.15 and conventional MAPbI_3_ are depicted in Figure 5b, showing negligible hysteresis regarding MAPbI_3_‐based perovskite solar cell, which is favorable for practical solar cell application. Figure 5c exhibits the ultraviolet–visible (UV–vis) absorption spectra of (FAPbI3)_1–*x*_ (MAPbBr_3_)*_x_* annealed at 100 °C with *x* = 0 (dark yellow curve), 0.05 (red curve), 0.15 (green curve) and 0.25 (blue curve). The black curve is FAPbI_3_ annealed at 150 °C and these results elucidate that with the increasing of *x* value, absorption band edge will shift to shorter wavelength, which is consistent with the external quantum efficiency (EQE) spectra in Figure 5d. It is found that MAPbBr_3_ facilitated the stability of FAPbI_3_ and this modulation of perovskite composition resulted in perovskite solar cells with an outstanding PCE of 18%.^37^

Subsequently, FAPbI_3_‐based perovskite solar cell with the highest PCE of 20.1% to date has been achieved by the same group (e.g., Seok et al.). Herein, inspired by study of Jeon et al. that PbI_2_–DMSO–MAI can be converted into MAPbI_3_,^8^ Seok and his colleagues proposed that DMSO molecules in PbI_2_–DMSO were also able to be simply exchanged via FAI molecules due to stronger interaction of FAI–PbI_2_ (ionic interaction) compared to DMSO–PbI_2_ (molecular interaction). They utilized this intramolecular exchange method and prepared superior FAPbI_3_ perovskite layer with large grain sized morphology and prior crystallization along (111) crystal orientation, thus resulting in corresponding perovskite solar cell with the highest PCE to date.^9^

Moreover, precursor engineering was feasible to tune morphology and crystallization of the perovskite layer. Using HPbI_3_ as precursor has been demonstrated to efficiently remove water of precursor solution and decrease crystallization rate of perovskite layer. Employing this novel precursor HPbI_3_ (not conventional PbI_2_) to react with FAI leads to the formation of homogeneous and stable light absorber and attains an average PCE of 15.4% in the perovskite solar cells.^38^ Incorporating polymer poly(ethyleneglycol) (PEG) as additives into precursor is probed by Chang and his colleagues. They added 1wt% PEG in perovskite layer and found that this process was also efficient in controlling morphology and crystallization. The additive PEG slows the crystal growth rate of perovskite layer, resulting in pinhole‐free and homogeneous film and enhancing PCE, current density (*J*_sc_) and *V*_oc_ of devices.^39^

In architecture of perovskite solar cell, perovskite layer plays a vital role in light absorption as well as charge carriers generation. In other words, controllable morphology and crystallization of perovskite layer will facilitate photovoltaic performance of subsequent devices. Therefore, achieving high performance perovskite solar cells has concentrated on exploring controllable methods to prepare high surface coverage, uniform, pin‐hole free and compact perovskite film. In this regard, thermal deposition and annealing, gas‐assisted methods, composition, solvent and precursor engineering have been investigated and demonstrated to be promising for high performance devices. However, for future improvements, these techniques are still essential to be improved. For example, thermal deposition and annealing should be further studied to process in lower temperature and simultaneously realize perovskite solar cells with decent performance. More novel gas‐assisted methods are also promising to obtain highly homogeneous perovskite film for future high performance devices. As MAPbI_3_ perovskite has been changed to FAPbI_3_ showing good performance, other strategies of composition engineering should be further investigated to discover innovative perovskite materials. Towards solvent and precursor engineering, apart from conventional DMF and DMSO, other similar additives are expected to optimize morphology and crystallization of perovskite layer, thus achieving devices with higher PCE.

## 3 HTMs

### 3.1 Organic HTMs

In order to extract and transport the holes generated by perovskite absorber, HTMs are widely studied towards realization of high performance perovskite solar cells. General considerations for choosing HTMs include: appropriate HOMO for efficient electron blocking and hole collection, high hole mobility, stable thermal and optical properties, along with the ability to be doped.^40^ Hence, novel HTMs are expected to be intensely explored and improve the photovoltaic performance of devices.^41^

**Figure 6:**
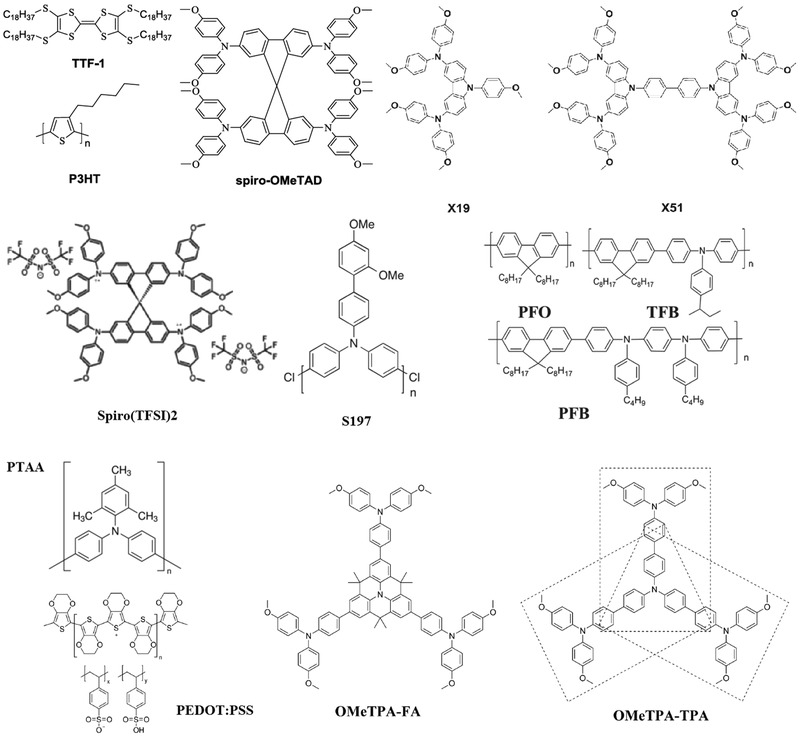
Molecular structures of organic HTMs including spiro‐OMeTAD, PFB, PFO, TFB. Reproduced with permission.^40^ Spiro(TFSI)2. Reproduced with permission.^45^ Copyright 2014, American Chemical Society. TTF‐1, P3HT. Reproduced with permission.^49^ Copyright 2014, Royal Society of Chemistry. S197. Reproduced with permission.^51^ X19, X51. Reproduced with permission.^53^ PTAA, PEDOT:PSS, OMeTPA‐FA and OMeTPA‐TPA. Reproduced with permission.^55^

State‐of‐the‐art organic HTM 2,2′,7,7′‐tetrakis (N,N‐dip‐p‐methoxypheny‐am ine)‐ 9,9′‐spirobifluorene (spiro‐MeOTAD; molecular structure shown in **Figure** **6**) has already emerged and paved the way to achieve high performance perovskite solar cell in a very short period.^42^ However, spiro‐MeOTAD suffers from low hole mobility and conductivity,^43^ owing to its pristine, unique structure. Additives, such as Li‐bis(trifluoromethanesulfonyl) imide (Li‐TFSI), perfluoro‐tetracyanoquino‐dime thane (F4TCNQ) and tris(2‐(1H‐ pyrazol‐1‐yl) pyridine) cobalt(III) (FK102 Co(III)) are necessary to dope and improve conductivity of spiro‐MeOTAD,^43^, ^44^ rendering high‐cost and complex synthesis in corresponding perovskite solar cell fabrication. To mitigate these problems, 2,2′,7,7′‐tetrakis (N,N‐di‐p‐methoxyphenylamine)‐9,9′‐spiro bifluorenedi [bis‐(trifluorom thanesulfonyl) imide] (spiro(TFSI)2; molecular structure is shown in Figure 6) has been studied, which enhances hole conductivity of spiro‐MeOTAD in inert atmosphere instead of oxidation in air. While spiro(TFSI)2 is similar to spiro‐MeOTAD in structure, no additives are necessary to pretreat spiro(TFSI)2 and subsequent perovskite solar cell is fabricated in inert atmosphere, which prevents degradation during device fabrication. As a result, spiro(TFSI)2‐based perovskite solar cells show almost the same PCE and better stability than spiro‐MeOTAD‐based devices, indicating their potential in practical application.^45^

**Figure 7:**
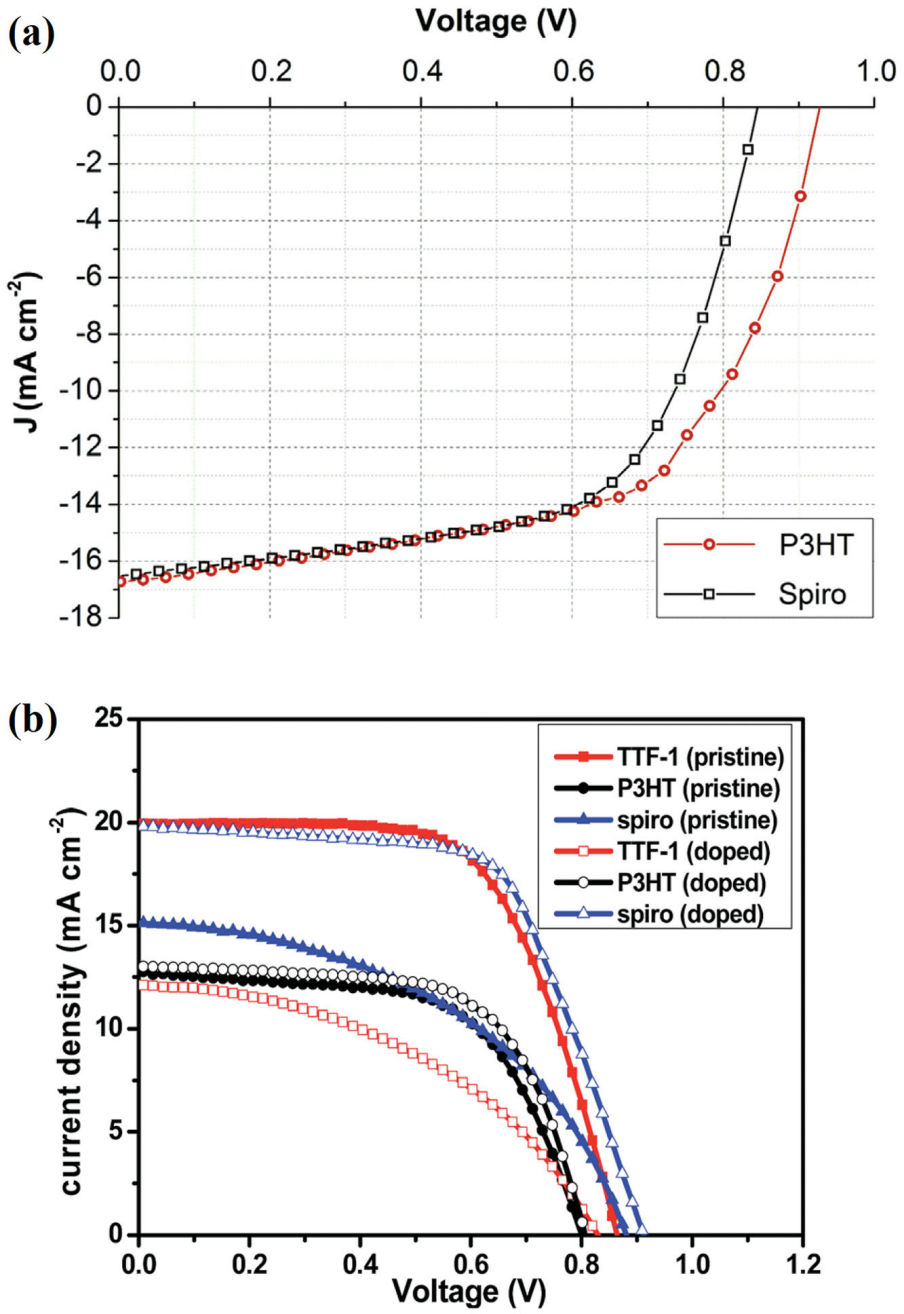
a) *J–V* curves of spiro‐MeOTAD‐based (black curve) and P3HT‐based devices (red curve) devices. Reproduced with permission.^47^ Copyright 2014, Elsevier. b) *J–V* curves of original and Li‐TFSI, TBP doped TTF‐1, P3HT and spiro‐MeOTAD based‐perovskite solar cells. Reproduced with permission.^49^ Copyright 2014, Royal Society of Chemistry.

Poly (3‐hexylthiophene‐2,5‐diyl) (P3HT, molecular structure is shown in Figure 6) has also been demonstrated as a comparably cost‐effective and high mobility HTM with respect to spiro‐MeOTAD in solar cells.^46^ Herein, a perovskite solar cell configuration of Au/P_3_HT/MAPbI_3–*x*_Cl*_x_*/TiO2/FTO was fabricated and studied by Giacomo et al.. They utilized spiro‐MeOTAD in the same device as HTM for comparison and further measured the *J–V* curves and discussed this HTM strategy, as illustrated in **Figure** **7**a. Figure 7a shows the *J–V* curve of spiro‐MeOTAD‐based and P3HT‐based devices, the black curve represents spiro‐MeOTAD HTM and red curve represents P3HT HTM, respectively. These two curves are almost identical except for higher *V*_oc_ of P3HT device due to an appropriate energy level, rendering a higher PCE (9.3%) than device fabricated with spiro‐MeOTAD (8.6%).^47^ Another work also elucidated the enhancement of photovoltaic parameters (such as *V*_oc_, fill factor (FF) and PCE) with P3HT HTM and higher stability of as‐fabricated device, confirming its crucial impact on perovskite solar cells.^48^

Compared with HTM P3HT and spiro‐MeOTAD, an original tetrathiafulvalene ‐based HTM TTF‐1(molecular structure shown in Figure 6) is investigated in perovskite solar cells. Chemical p‐type doping is unnecessary in HTM TTF‐1 with respect to complex pretreatments of HTM spiro‐MeOTAD, appropriate HOMO level and unique molecular structure of TTF‐1 give rise to high hole conductivity and mobility. Corresponding *J–V* curves of original and Li‐TFSI, TBP doped TTF‐1, P3HT and spiro‐MeOTAD based‐perovskite solar cells are shown in Figure 7b. A PCE of 11.03% with undoped TTF‐1 is achieved while undoped P3HT and spiro‐MeOTAD only show a PCE of 6%. Moreover, Li‐TFSI/TBP‐doped P3HT and spiro‐MeOTAD‐based device just attained a PCE of 6.72 and 11.4%, respectively. Further characterization of corresponding devices exhibited better stability of TTF‐1 as compared with doped spiro‐MeOTAD, which stems from long alkyl chains in TTF‐1, thus promoting the improvement of undoped and stable HTMs in perovskite solar cells.^49^

**Figure 8:**
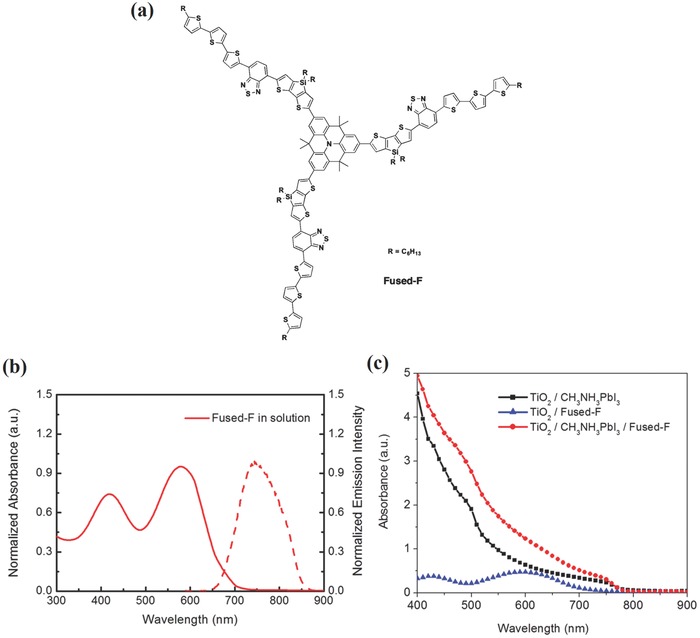
a) Molecular structure of Fused‐F. b) Absorbance (UV–vis spectra) and emission intensity (fluorescence spectra) of Fused‐F (in chloroform solution). c) UV–vis spectra of TiO_2_/Fused‐F, TiO_2_/MAPbI_3_ layers and TiO_2_/MAPbI_3_ layers/Fused‐F. Reproduced with permission.^50^ Copyright 2014, American Chemical Society.

As dopant‐free TTF‐1 is proved to be an efficient HTM, another dopant‐free quinolizinoacridine‐based HTM (Fused‐F, molecular structure is shown in **Figure** **8**a) is prepared by Qin et al. In this study, 4‐[5‐bromo‐3,3′‐dihexylsilylene‐2,2′‐ bithiophene] ‐7‐[5″‐n‐hexyl‐(2,2′;5′,2″‐terthiophene)‐5‐yl]‐benzo[c]‐[1,2,5]thiadiazole groups are utilized to achieve narrow band gap and planar, star‐shaped architecture, resulting in an appropriate energy level and outstanding hole transport capacities. Considering that Fused‐F is a colored HTM, UV–vis and fluorescence spectra in chloroform solution are measured, as illustrated in Figure 8b. Solid line exhibits that Fused‐F has strong absorptions in range of the visible light wavelengths (peak at 421 and 580 nm), which seems to harvest the light through perovskite layer, as well, emission peak (dash line) at 746 nm is observed in fluorescence spectrum. UV–vis spectra of TiO_2_/Fused‐F and TiO_2_/MAPbI_3_ layers/ Fused‐F(with or without) is characterized and exhibited in Figure 8c, elucidating that MAPbI_3_ layers with Fused‐F have stronger absorptions than MAPbI_3_ layers without Fused‐F, thus further proving the role of Fused‐F in extra light harvesting. In addition, Fused‐F‐based perovskite solar cell shows a PCE of 12.8% under 98.8 mW cm^−2^ (not standard illumination), which is higher than spiro‐MeOTAD‐based perovskite solar cell (a PCE of 11.7%) in the same configuration.^50^ Oligomer HTM S197 (molecular structure shown in Figure 6) is investigated as well by this group, which possesses low molecular weight, reasonable hole mobility, proper HOMO and cost‐effective properties, thus resulting in a device with a considerable PCE of 12% and providing new strategy for oligomer HTMs employed in high performance perovskite solar cell.^51^

Meanwhile, organic derivatives have been studied by plenty of groups. Carbazole‐based derivatives are considered as capable HTMs due to their good charge‐transport ability and facile modulation of optoelectronic properties by replacing carbazole group with other functional groups.^52^ Herein, small‐molecule carbazole‐based HTMs (abbreviated as X19 and X51, molecular structures are shown in Figure 6) are fabricated to construct perovskite solar cells by a simple synthesis approach. Further characterization illustrates that X51‐based perovskite solar cell presents a higher PCE (9.8%) than X19‐based device (7.6%), which was competitive in regard to spiro‐MeOTAD‐based device (10.2%), manifesting that these two small‐molecule carbazole‐based derivatives are promising HTMs in perovskite solar cells. More importantly, composition engineering of organics has been demonstrated as an efficient method to realize HTMs with high hole conductivity and mobility.^53^

Organic polyfluorene derivatives PFB, TFB and PFO (molecular structures are shown in Figure 6) are also probed by Zhu et al.. In this investigation, the HOMO of PFO is found to be –5.8 eV, which is lower than MAPbI_3_, resulting in inefficient hole transport. Hole mobility of PFB, TFB and PFO are measured to be higher than spiro‐MeOTAD. To further confirm the advantages of these three organic derivatives, corresponding devices, including a spiro‐MeOTAD‐based referential device, are fabricated by one‐step and sequential processes. In one‐step processed devices, PFB, TFB and PFO show a PCE of 8.03%, 10.92% and 1.22%, respectively. Here, TFB‐based device is highlighted for the highest PCE even better than spiro‐MeOTAD‐based device (9.78%). As for sequential processed devices, TFB‐based device still holds a PCE of 12.8% comparable to spiro‐MeOTAD‐based device, showing that TFB is an efficient HTM for high performance perovskite solar cells.^40^

Moreover, Jeon et al. investigated HTM N, N‐di‐p‐methoxyphenylamine‐sub stituted pyrene derivatives that are low cost and able for fast charge‐transport. As a result, a considerable 12.4% PCE (12.7% PCE for spiro‐MeOTAD) is obtained in an as‐fabricated perovskite solar cell, thus indicating that pyrene arylamine derivatives are promising HTMs to replace traditional spiro‐MeOTAD.^54^ Tris(N,N‐bis (4‐methoxyphenyl)‐N‐phenylamine)quinolizinoacridine(OMeTPA‐FA) and tris(N,N‐ bis(4‐methoxyphenyl)‐N‐biphenyl)amine (OMeTPA‐TPA) (molecular structures shown in Figure 6) are prepared as HTMs and achieve perovskite solar cells with a PCE more than 13%. However, complex pretreatments are still necessary in these HTMs preparation.^55^

Employing PEDOT:PSS (molecular structure is shown in Figure 6) as HTM in perovskite solar cells has been demonstrated to be feasible as well. In general device architectures with HTM PEDOT:PSS, the perovskite layer is sandwiched between HTM PEDOT:PSS and ETM [6,6]‐phenyl‐C61‐butyric acid methyl ester (PCBM). Perovskite solar cells with such structure are able to reach PCE of 11.5%, 14.1% and even 18%, showing that PEDOT:PSS is an outstanding HTM in perovskite solar cells.^56^, ^57^ Exploiting poly[bis(4‐phenyl) (2,4,6‐trimethylphenyl) amine](PTAA, molecular is shown in Figure 6) in perovskite solar cells is another strategy to achieve a high PCE in perovskite solar cells, which stems from its considerable hole mobility and suitable HOMO level in terms of perovskite layer, resulting in a prevalent trend to use PTAA as HTM for high performance perovskite solar cells with a superior PCE over 16%.^8^, ^37^, ^56^, ^58^

For organic HTMs, although commonly used spiro‐MeOTAD is expensive and necessitates extra treatments such as doping, it is still prevalent in perovskite solar cells. Plenty of organic HTMs such as P3HT, TTF‐1, PTAA and PEDOT:PSS have been studied and exhibit considerable device performance. Nevertheless, improvements of organic HTMs are still essential for future high performance perovskite solar cells. First, further device physics such as charge recombination of HTM/perovskite layer contact should be understood, more organic HTMs with appropriate HOMO relative to perovskite layer should be studied, which enhance the ability of hole extraction and transport, resulting in devices with better photovoltaic performance. Second, low cost organic HTMs without additional treatments and possess high hole mobility are expected in prospective devices. Third, approaches regarding organic molecular designing and engineering should be explored, which produce more unique organic derivatives as efficient organic HTMs for high performance perovskite solar cells. In addition, HTMs with extra light absorption are promising to improve photovoltaic performance of future devices.

### 3.2 Inorganic HTMs

In contrast with commonly expensive organic HTMs, which hinder the commercial applications of perovskite solar cells, inorganic HTMs have triggered widespread interests. In this regard, CuI, with reasonably high hole conductivity and facile preparation, is studied by Christians et al.. Here, CuI is deposited on MAPbI_3_/TiO_2_ by solution method and the perovskite solar cell structure is Au/CuI/MAPbI_3_ /TiO2/FTO. At the same time, spiro‐MeOTAD is used as HTM in identical device architecture for comparison. The CuI‐based device has reached PCE of 6%, which is comparable to spiro‐MeOTAD‐based devices (the highest PCE is 7.9%). Impedance spectroscopy further elucidates higher electrical conductivity and recombination rate in CuI‐based device, rendering higher FF and lower *V*_oc_ as compared to reference spiro‐MeOTAD‐based device. Moreover, the CuI‐based device is more stable than spiro‐MeOTA‐based device under illumination.^59^

CuI‐based perovskite solar cell shows a relatively low PCE mainly due to its inappropriate energy level against perovskite layer. Then, NiO, another p‐type inorganic oxide, which performs well in former solar cells,^60^ has been intensively investigated by several groups. Subbiah et al. used electrodeposition technique to prepare NiO as inorganic HTM in Ag/PCBM/MAPbI_3–*x*_Cl*_x_*/NiO/FTO perovskite solar cell and fabricated the same architecture of CuSCN‐based device for comparison. They achieved a PCE of 7.3% and 3.8% for HTM NiO and CuSCN, respectively. This work provides a promising strategy for NiO and CuSCN as inorganic HTM in perovskite solar cells, which leads to further studies and optimization.^61^

**Figure 9:**
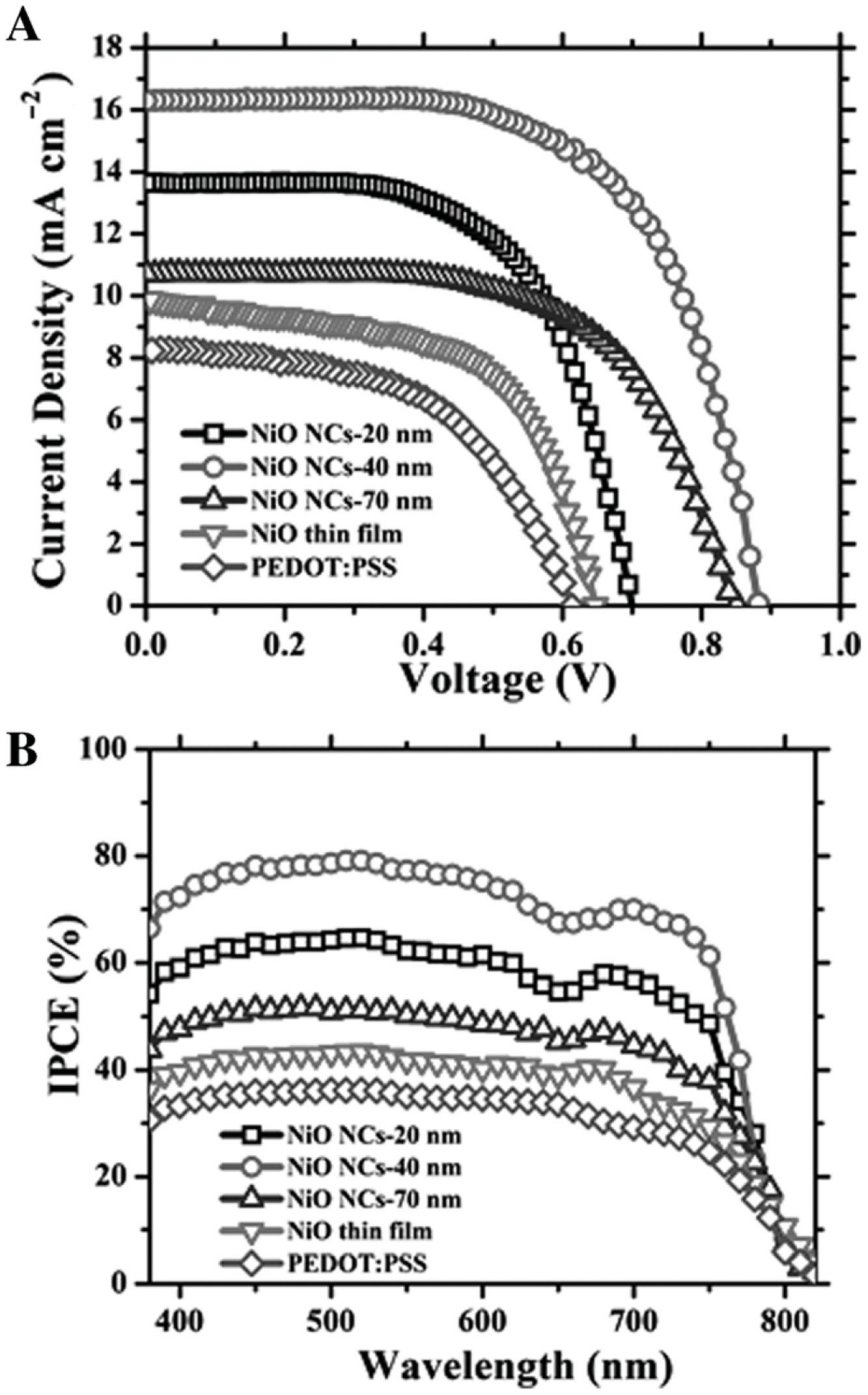
a) *J–V* curves of perovskite solar cells employing NiO NCs (20 nm, 40 nm and 70 nm), thin film and organic PEDOT: PSS as HTMs. b) Corresponding IPCE spectra of NiO NCs (20 nm, 40 nm and 70 nm), thin film and organic PEDOT: PSS‐based perovskite solar cells. Reproduced with permission.^62^

For other inorganic HTMs based on nickel oxide, NiO nanocrystal and film prepared by facile sol‐gel‐approach have been utilized in perovskite solar cells and the impact of film thickness on device performance is studied, as depicted in **Figure** **9**. Figure 9a illustrates *J–V* curve of as‐fabricated devices using various thicknesses (20 nm, 40 nm and 70 nm) of NiO nanocrystals (NiO NCs), thin film and organic PEDOT: PSS as HTMs. High PCE of 9.11% is accomplished in 40 nm NiO NCs‐based device in comparison with 20 nm NiO NCs‐based device (PCE of 6.04%) and 70 nm NiO NCs‐based device (PCE of 5.58%), which is superior to NiO thin film and organic PEDOT: PSS‐based devices. PCE enhancement of NiO NCs‐based device (NiO NCs thickness change from 20 nm to 40 nm) is attributed to decrease of charge recombination, leakage current and increase of electron blocking. On the other hand, improved series resistance and photoabsorption (NiO NCs thickness change from 40 to 70 nm) cause the degraded device performance. The incident‐photon‐to‐current efficiency (IPCE) spectra is shown in Figure 9b, confirming the optimum performance of the 40 nm NiO NCs‐based device.^62^ Moreover, configuration of NiO/MAPbI3/[6,6]‐phenyl C61‐butyric acid methyl ester (PC61BM) has been fabricated using mesoscopic NiO as HTM, which renders more loading of perovskite material due to its natural scaffold, thus leading to a PCE of 9.51%.^63^

**Figure 10:**
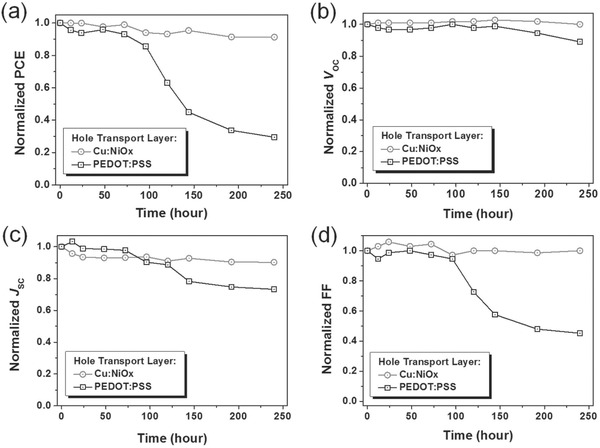
Time‐dependent normalized photovoltaic parameters of PEDOT:PSS and Cu:NiO*_x_* –based perovskite solar cells including a) PCE, b) *V*_oc_, c) *J*_sc_ and d) FF. Reproduced with permission.^64^

A novel doping method for NiO*_x_* by using Cu as dopant is proposed by Kim and co‐workers.^64^ Here, PEDOT: PSS and NiO*_x_* film are compared. Perovskite layer MAPb(I_1–*x*_Br*_x_*)_3_ with wide band gap is utilized to highlight the potential application of HTM Cu‐doped NiO*_x_* in multijunction solar cells. The appropriate amount of Cu additive (5 at%) is verified to improve electronic conductivity of NiO*_x_* by current‐ voltage curve, which results in the enhancement of photovoltaic parameters including *V*_oc_, *J*_sc_ and FF, further reducing the series resistance. Photoluminescence (PL) spectra exhibit evident quenching of Cu‐doped NiO*_x_*, showing more effective charge collection. SEM images depict that perovskite layer grown on Cu‐doped NiO*_x_* tends to have larger grain size, which is favorable for high performance perovskite solar cell, thus attaining a PCE of 15.4%. Moreover, stability of Cu‐doped NiO*_x_*‐based device in ambient is measured along with PEDOT: PSS‐based device as exhibited in **Figure** **10**. Figure 10a–d clarify the normalized time‐dependent photovoltaic parameters including PCE, *V*_oc_, *J*_sc_ and FF. The PCE of PEDOT: PSS‐based device decreases quickly with the reduced *J*_sc_ and FF after about 100 h in ambient while Cu‐doped NiO*_x_*‐based device keep a stable PCE even after 10 days, thus manifesting that this doping strategy is efficient in achieving long‐term stable and high performance perovskite solar cells.^64^

In addition, Wu and co‐workers investigated perovskite solar cell based on HTM of inorganic graphene oxide (GO). *J–V* curve of device based on 2 nm GO HTM exhibited a high PCE of 12.4%, which was derived from the efficient hole extraction of GO and its effect on promoting uniform perovskite layer formation and optimizing surface coverage.^17^ Unlike mentioned above that Ag/PCBM/MAPbI_3–*x*_Cl*_x_*/CuSCN /FTO perovskite solar cell only possesses a low PCE of 3.8%, it has been proved by Qin et al. that CuSCN is actually an encouraging inorganic HTM in perovskite solar cells. In this study, the device structure is Au/CuSCN/MAPbI_3_/TiO2/FTO and MAPbI_3_ layer is deposited once or twice to compare the light‐harvesting. The performance of corresponding perovskite solar cells is investigated with and without HTM CuSCN, which is prepared by doctor blading process. For once deposited devices, PCE of 11.2% and 3.8% are attained with and without CuSCN, respectively. On the other hand, twice deposited devices with and without CuSCN shows a higher PCE of 12.4% and 6.7%, elucidating that twice deposition of MAPbI_3_ layer is efficient for improvement of device PCE. Most importantly, inorganic HTM CuSCN is verified to be favorable in realizing high performance perovskite solar cell.^65^

Investigations of inorganic HTMs are promising for high performance perovskite solar cells due to their natural low cost and stable properties compared to organic HTMs. However, devices with inorganic HTMs suffer from over‐speed charge recombination rate and lower photovoltaic parameters (such as FF). For future developments, inorganic HTMs with proper energy‐level alignment contacted with perovskite layer is crucial, at the same time, charge recombination should be optimized and family of inorganic HTMs for high performance perovskite solar cells is expected to expand. More doping techniques of inorganic HTMs should also be investigated to improve device performance. Besides, highly durable inorganic HTMs that can boost achievement of prospective long‐term stable and high performance perovskite solar cells should be further explored.

## 4 ETMs

### 4.1 Nanostructured ETMs

Consistent with HTMs, ETMs between perovskite layer and transparent electrode (ITO/FTO) necessitate proper CBM for effective electron extraction and hole blocking. In mesoscopic perovskite solar cells, ETMs also act as scaffold to achieve perovskite deposition. Different from conventional mesoporous ETMs, nanostructured ETMs on ITO/FTO possess advantages offering sufficient perovskite materials filling and favorable electron transportation,^66^ thus enhancing performance of perovskite solar cells. Meanwhile, nanostructured ETMs can be prepared via solvent‐thermal and hydrothermal techniques,^67^ which prevent high temperature sintering of conventional compact TiO_2_/ZnO ETMs, resulting in lower device costs and potential for commercialization.

Mesoporous TiO_2_ is commonly applied as scaffold and ETM for high performance perovskite solar cells due to its appropriate energy level and competent electron collection.^8^ However, poor efficiency of pore‐filling is proposed as an obstacle, leading to the explorations of novel nanostructures for developed perovskite solar cells. Aimed at TiO_2_ nanostructures as ETMs, Qiu et al. synthesized TiO_2_ nanowire arrays as ETM and a considerable perovskite solar cell (with a PCE of 4.87%) was achieved in early 2013. The morphology parameters of these TiO_2_ nanowire arrays were investigated, showing that small‐diameter and dense TiO_2_ nanowire arrays are favorable for high performance devices.^68^ Also, TiO_2_ nanotube arrays were formed on Ti foil as ETM and accomplished a PCE of 8.31% in assembled perovskite solar cell. Such solar cell is also flexible with combination of Ti foil‐based substrate and TiO_2_ nanotube arrays. However, the TiO_2_ nanotube arrays necessitate a high temperature annealing to enable improved electron transport, which is not cost‐effective.^69^ Furthermore, TiO_2_ nanorod was considered as a promising ETM because of its superior electron transport in solar cells.^70^ To this end, Kim et al. proposed the employment of TiO_2_ nanorod in perovskite solar cell and found that uniformly ordered and short TiO_2_ nanorod‐based layer was desirable as ETM to realize high performance perovskite solar cell (a PCE of 10%).^71^ To avoid environmental issues, TiO_2_ nanorods are also prepared without strong acid by hydrothermal technique and fabricated as efficient ETM to complete perovskite solar cell with a PCE of 11%. Again, the device tends to behave well with shorter length of nanorod, which is consistent with investigation of Kim et al., thus confirming that the TiO_2_ nanorod is a capable scaffold material and ETM in perovskite solar cells.^72^

TiO_2_ nanoparticles are probed to be an efficient ETM in solar cells.^73^ In this vein, Yella and co‐workers developed a low temperature chemical bath deposition method to prepare a TiO_2_ nanoparticle layer as ETM, which creates a close connection with perovskite layer, showing good electron transport property. This connection is emphasized to produce large area contact with light harvester, which is suitable for electron extraction, giving rise to a perovskite solar cell with a PCE of 13.7% and high *V*_oc_ over 1 V.^74^ Wang et al. also prepared TiO_2_ nanoparticles with additive graphene nanoflakes by a low temperature solution processed method. The as‐prepared nanocomposite revealed that graphene nanoflake played a critical role in enhancing electron collection and transport in ETM owing to its extraordinary conductivity, which reduced series resistance in corresponding perovskite solar cells, and thus realized high PCE reaching up to 15.6%.^75^ Additionally, TiO_2_ nanoparticles immersing in an alcoholic solvent with TiAcAc without high temperature sintering is demonstrated to yield compact ETM. The improved conductivity and reduced series resistance result in a perovskite solar cell with outstanding PCE of 15.9%.^76^ Unlike conventional ETMs prepared by high temperature sintering technique, low temperature solution processed TiO_2_ nanoparticle‐based ETMs are promising in flexible devices and tandem solar cells.

Moreover, innovative and well‐controlled TiO_2_ nanocones‐based ETM is synthesized by Zhong et al. with an environment friendly and low temperature hydrothermal process. Time‐resolved PL spectroscopy elucidates that electron transport of TiO_2_ nanocones‐based ETM outperforms TiO_2_ nanorods‐based ETM, which may stems from the higher surface‐to‐volume ratio and axial electrostatic variation of TiO_2_ nanocones as compared to TiO_2_ naonrod. Therefore, reduced charge recombination and high performance perovskite solar cell with a PCE of 11% is realized.^77^

Apart from TiO_2_ nanostructures, ZnO nanostructures are examined and used as ETMs in perovskite solar cells. ZnO nanorods‐based ETM prepared by low temperature chemical bath deposition was confirmed to enhance *J*_sc_ in as‐fabricated perovskite solar cell, which is attributed to improved charge generation and collection with unique nanorod morphology, rendering notable PCE of 8.9% for inflexible substrate and a PCE of 2.62% for flexible devices.^78^ Son et al. utilized different solution techniques and prepared ZnO nanorods anchored on a ZnO seed layer as ETM, here, the morphology of ZnO nanorods could be manipulated via precursor and growth condition. Assembled device attains a competitive PCE of 11% and the photocurrent response of the referential TiO_2_ nanorods‐based ETM with consistent device architecture reveals a lower electron collection rate than ZnO nanorods‐based ETM, exhibiting that ZnO nanorods‐based ETM is promising in electron extraction and transport in perovskite solar cell.^79^ In addition, magnetron sputter process is employed to prepare oriented ZnO nanorods‐based ETM, which also gives rise to well electron transport property with well‐aligned nanorods along c‐axis. The photovoltaic parameters are sensitive with various thickness of ZnO nanorods‐based ETM and corresponding devices exhibit a high PCE of 13.4% and 8.03% for rigid and flexible substrates, respectively. Considering the commonly used magnetron sputter approach in commercial application, it holds the promise in future large‐scale perovskite solar cells.^80^

Liu and co‐workers proposed a solution‐processed method and prepared an ultrathin ZnO nanoparticle layer, which was fabricated as ETM in perovskite solar cell without high temperature sintering. Devices using the ultrathin ZnO nanoparticle layer as ETM no longer require scaffold materials, which allows for ideal perovskite layer crystallization without any constrains, thus optimizing effective optical pathway and forming high performance, inflexible and flexible perovskite solar cell with PCE of 15.7% and 10.2%, respectively.^81^

Electrochemical deposition method, which is able to manipulate nanostructure morphology in low temperature, has also been developed to prepare nanostructured ZnO layer as ETM in perovskite solar cell. Introducing a low n‐type doped ZnO as shell to cover as‐prepared ZnO nanowires and nanorods layer is realized by high over‐voltage electrochemical deposition, which enhances the *V*_oc_ and *J*_sc_ of subsequent solar cell owing to the suppressed charge recombination, resulting in a considerable PCE of 10%. This work manifests that low temperature electrochemical deposition is a favorable process to precisely control formation of nanostructured ETM and its surface modifications.^82^

Moreover, bilayer ZnO nanostructures are suggested and studied by Mahmood et al.. They synthesized ZnO nanorods as an overlayer anchoring on an underlayer of ZnO nanosheet arrays and such nanostructured ZnO ETM was assembled in a perovskite solar cell. The density of ZnO nanorods can be well controlled by altering precursor concentration. A high nanorod density is able to enhance the PCE and *V*_oc_ of as‐fabricated perovskite solar cell. These improved photovoltaic parameters are mainly attributed to the optimal electron collection and transport caused by large area contact between perovskite light harvester and bilayer ZnO ETM. However, extremely high concentration of precursor will cause reduced photovoltaic performance, which is related to mismatched energy band gap of nanorods and perovskite layer. ZnO nanosheet arrays provide better loading of perovskite layer and bilayer nanostructured ZnO as ETM performs well in corresponding perovskite solar cells with decent stability and PCE as high as 10.35%.^83^

Aiming at electron extraction and transport, nanostructured ETMs have been widely investigated due to their advantages of morphology and construction in tuning their own electronic properties, which in turn affect photovoltaic performance of as‐fabricated devices. Conventional ETMs such as TiO_2_ and ZnO have been prepared into various nanostructures improving device performance and low‐temperature approaches for nanostructures preparation are dominant. However, other potential ETMs like nanostructured SnO2 and CdS should also be studied. Except for regular nanostructured ETMs like nanorod, nanoparticle and nanowire‐based ETMs, other nanostructures such as nanosphere should be attempted to improve device performance. As morphology and surface coverage of nanostructures is able to affect consequent device performance, more controllable methods are required for prospective preparation of nanostructured ETMs. Apart from additives such as nanostructured graphene, other additives that can enhance electron mobility of ETMs, are favorable and should be further investigated to achieve future perovskite solar cells close to theoretical efficiencies.

### 4.2 ETM Modification

Regardless of diverse nanostructured ETMs, enormous studies have focused on ETMs modification in order to enhance performance of perovskite solar cells. In this regard, Zhu et al. introduced a graphene quantum dots (GQDs) layer (several nanometers) upon TiO_2_ ETM by electrochemical technique. Further investigations indicated that the ETM with GQDs layer performed superior than ETM lacking GQDs layer when incorporated in perovskite solar cells with the same structures. That is, the GQDs layer‐based perovskite solar cell held a PCE as high as 10.15% while the device without GQDs layer simply exhibited a PCE of 8.81%. Ultrafast transient absorption spectroscopy elucidated that GQDs layer‐based device was able to extract electron in a shorter time (90–106 ps) than device without GQDs layer (260–307 ps), thus resulting in an improved PCE. The GQDs layer here act as a capable electron pathway to modify ETM TiO_2_, providing a significant enlightenment for designing prospective perovskite solar cell.^84^

Employing polyelectrolyte layer to modify ETM has been studied by Brabec's group. Herein, polyethyleneimine ethoxylated (PEIE) and poly[3‐(6–trimethylammonium hexyl) thiophene] (P3TMAHT) are fabricated between PCBM (ETM) and Ag electrode. The PCE of as‐fabricated devices increases from 8.5% to 12% (based on PEIE) and 11.28% (based on P3TMAHT), respectively. It is demonstrated that the utilization of these polyelectrolyte layers result in additional surface dipole at PCBM/Ag interface, which lowers the work function of metal electrode and provides easier electron injection into ETM, further enhancing performance of subsequent perovskite solar cells.^85^ In addition, this group has investigated organic derivatives for ETMs modification. An amine‐functionalized fullerene derivative named DMAPA‐C60 is incorporated between ETM PC60BM and Ag electrode in perovskite solar cell with architecture of Ag/PC60BM/perovskite layer/PEDOT:PSS/ITO. As‐prepared DMAPA‐C60 interlayer in these devices gives rise to reduced work function of metal electrode, better surface morphology of perovskite layer and optimized energy‐level alignment between ETM and perovskite absorber, resulting in notable improvements of PCE (from 9.4% to 13.4%) and FF (61% to 77%).^86^ Analogous to DMAPA‐C60, another perylene−diimide derivative (PDINO) has also been fabricated at interface of ETM‐metal electrode in perovskite solar cells, which is able to effectively modify the ETM. Similarly, the PDINO interlayer leads to decreased work function of Ag electrode, enhanced electron collection and lower shunt resistance, thus improving PCE (from 10% to 14%) in perovskite solar cells.^87^

Al‐doped ZnO (AZO) has been proved to own proper energy level, improved electron mobility and density than ZnO.^88^ Therefore, Dong and co‐workers assembled perovskite solar cells with AZO modified ZnO nanorods. AZO was deposited on ZnO nanorods like a shell via spin‐coating process and reduced charge recombination at perovskite layer/ETM interface, which was demonstrated by impedance spectroscopy, further giving rise to an enhancement of PCE (from 8.5% to 10.07%) and *V*_oc_ when compared with device without AZO modification. This improvement is owing to the optimization that AZO provides a buffer regarding conduction band offset of perovskite layer and ZnO nanorod‐based ETM and doping of AZO results in increased electron density of ZnO nanorod‐based ETM.^89^

**Figure 11:**
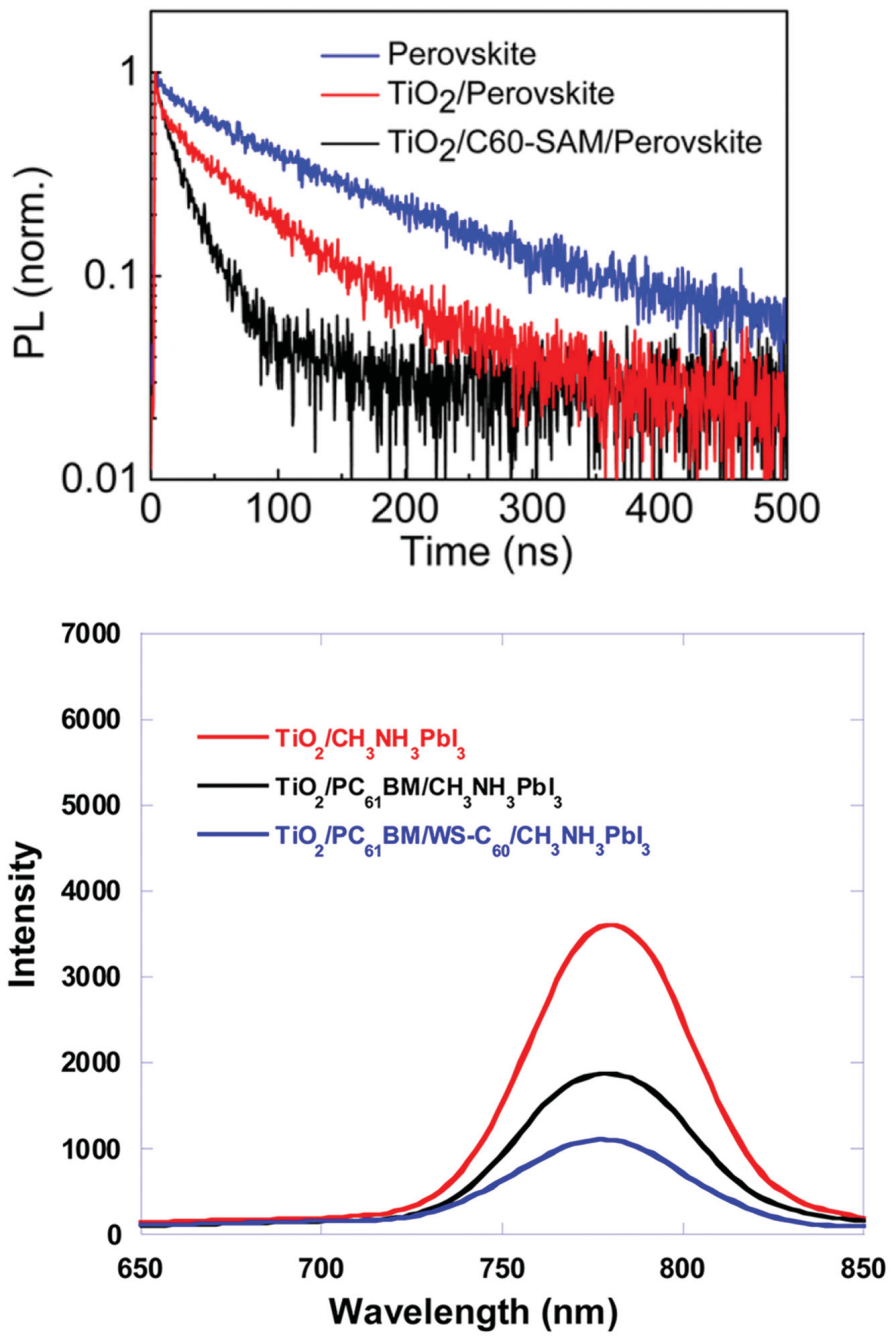
a) Time‐resolved PL decays of perovskite, TiO_2_/perovskite and TiO_2_/C_60_‐SAM/perovskite. Reproduced with permission.^90^ Copyright 2014, American Chemical Society. b) PL spectra of TiO_2_, TiO_2_/ PC_61_BM and TiO_2_/WS‐C_60_ modified PC_61_BM. Reproduced with permission.^92^ Copyright 2015, American Chemical Society.

An organic fullerene self‐assembled monolayer (SAM) additive abbreviated as C_60_‐SAM was studied concerning electron transport of ETM (TiO_2_) in corresponding perovskite solar cell. Time‐resolved PL decays were measured as illustrated in **Figure** **11**a. Figure 11a portrayed that the PL quenching effect of TiO_2_/C_60_‐SAM/perovskite was much stronger than TiO_2_/perovskite, showing a faster electron transport after C_60_‐SAM modification. Furthermore, group of C_60_‐SAM efficiently suppressed the trap states on TiO_2_ and charge recombination at perovskite interface, resulting in an enhanced PCE (11.5 to 14.8%) of as‐fabricated perovskite solar cell. It can be seen that prevalent trends will focus on developing novel organic additives with functional groups for optimizing ETM by reducing charge recombination and increasing electron transport.^90^ Organic silane SAM is also demonstrated to be promising in ETM modification, which suppresses charge recombination and optimizes electronic structure between ETM and perovskite layer, further giving rise to a high performance device with a PCE of 12.7%.^14^ Moreover, Zuo and co‐workers utilized 3‐aminopropanioc acid (C3‐SAM) to modify ETM (ZnO). It is found that C3‐SAM modification is favorable for morphology and crystallization of perovskite layer, which enhances light‐harvesting. In the meantime, work function of electrode is decreased for better charge carrier collection and electronic interaction at ETM/perovskite layer interface is enhanced, thus leading to a tremendous improvement of device PCE from 12% to 15.7%.^91^

Liu et al. utilized [6,6]‐phenyl‐C61‐butyric acid methyl ester (PC_61_BM) to fabricate perovskite solar cell because of its superior electronic conductivity than traditional ETM TiO_2_. However, perovskite layer was difficult to fully cover the surface of PC_61_BM. An additive fullerene derivative (WS‐C_60_) was employed to modify PC_61_BM to solve the issue of perovskite layer coverage. PL spectra of TiO_2_, TiO_2_/ PC_61_BM and TiO_2_/WS‐C_60_ modified PC_61_BM were shown in Figure 11b, showing a remarkable photoluminescence quenching phenomenon of ETM with and without PC_61_BM, confirming the improved electron transport and collection of PC_61_BM functionalized ETM. Stronger photoluminescence quenching phenomenon is observed with WS‐C_60_ modified PC_61_BM, elucidating the formation of more uniform perovskite layer on as‐modified ETM, thus leading to large J_sc_ and an enhancement of PCE from 13.2% to 14.6% with WS‐C_60_ modified PC_61_BM ETM in perovskite solar cells.^92^

Another significant ETM modification for high performance perovskite solar cell was studied by Zhou and co‐workers as well. Yttrium‐doping was used to modify ETM TiO_2_, which gave rise to an improvement of electron transport and extraction, thus enhancing the photovoltaic parameters of subsequent device including *J*_sc_ and PCE. Besides, an important modification of PEIE layer between ETM and ITO resulted in a reduced work function of ITO, which matched well with that of as‐modified ETM, consequently realizing a PCE as high as 19.3% in corresponding perovskite solar cell.^93^

ETMs modification, such as additional organic or inorganic layer at interface of ETMs and electrodes, has been demonstrated to effectively improve device performance. To further enhance photovoltaic performance, future ETMs modification should still focus on realizing better electron extraction and transport. That is, appropriate ETMs modification is expected to tune the energy level alignment between ETM and electrode, reducing recombination rate and even affecting perovskite layer morphology and crystallization, which are all vital factors for enhancing future device performance.

## 5 HTM and ETM‐Free Architectures

In order to further reduce the device costs, emerging studies have concentrated on HTM and ETM‐free perovskite solar cells. In these devices, perovskite layer with low exciton binding energy at room temperature acts as both light harvester and charge transport materials,^94^ rendering promising cost‐effective device architectures.

In 2012, Etgar and co‐workers proposed and probed the first HTM‐free perovskite solar cell with configuration of Au/MAPbI_3_ nanoparticle/TiO_2_ nanosheets. Herein, MAPbI_3_ played an important role both in light absorbing and hole transporting and subsequent device exhibits a reasonable PCE of 5.5%. Interestingly, a higher PCE of 7.3% was achieved under 100 W m^−2^ illumination (not standard AM 1.5 solar light). However, improved photovoltaic parameters are essential in this HTM‐free device.^95^

Based on identical device architecture (Au/MAPbI_3_/TiO_2_), Etgar et al. prepared an ultrathin TiO_2_ film to replace the TiO_2_ nanosheets and as‐fabricated HTM‐free perovskite solar cell attained a considerable PCE of 8%. Capacitance voltage measurements indicated that there existed a built‐in field of depletion, which facilitated charge separation and prevented electron dissipation from ETM to perovskite layer, further resulting in the enhancement of photovoltaic parameters.^96^

Besides, Etgar group improved the sequential spin coating process by considering the spin speed and wait time effects of spin coating, which produced high quality perovskite layer. XRD measurement revealed no change of crystallographic structure after one month, confirming the high stability of corresponding device, which showed a PCE as high as 10.85%.^97^

Inspired by the prevalent Au/MAPbI_3_/TiO_2_ architecture, Shi et al. fabricated HTM‐free perovskite solar cells via sequential deposition technique and examined the work principle, which is elucidated by ideal single heterojunction model (not sensitized solar cell). Further measurements of photovoltaic parameters also express a comparative PCE of 10.49% in terms of conventional thin‐film solar cells.^98^ In another similar work regarding HTM‐free perovskite solar cell, they developed the sequential method by reducing spin‐coating speed and increasing precursor and attained more homogeneous perovskite layer, which displayed an enhancement of light‐harvesting and charge carrier lifetime, thus obtaining a high PCE of 10.47%. Note that the as‐fabricated device holds a *V*_oc_ value as high as 0.95 V, showing outstanding photovoltaic parameters based on HTM‐free architecture.^99^

Wei and his colleagues also investigated HTM‐free perovskite solar cell by introducing a AlO*_x_* layer on MAPbI_3_ film, which blocked the electrons and improved charge extraction, giving rise to a high PCE of 11.10%.^100^ Most significantly, it is found that high performance HTM‐free perovskite solar cell can be achieved by altering the crystal structure of perovskite layer. Mei and co‐workers utilized a hybrid carbon/TiO_2_/ZrO_2_ layer as scaffold and modified MAPbI3 with 5‐ammoniumvaleric acid (5‐AVA) ions, which substituted MA site and formed a unique hybrid perovskite absorber named (5‐AVA)*_x_*(MA)_1–*x*_PbI_3_. It is demonstrated that the as‐prepared (5‐AVA)_x_(MA)_1–*x*_PbI_3_ absorber had fewer defects, optimal surface coverage and pore infiltration on scaffold as compared with MAPbI_3_ layer, thus leading to an enhanced PCE reaching up to 12.8% in subsequent HTM‐free device.^101^

Significantly, Li and his colleagues have fabricated an inverted HTM‐free perovskite solar cell showing a relatively high PCE of 16%. In this HTM‐free device, C_60_ was prepared as ETM on perovskite layer and 2,9‐dimethyl‐4,7‐diphenyl‐1,10‐ phenanthroline (BCP) was deposited on C_60_ acting as hole blocking layer, UV‐Ozone (UVO) treatment was also employed to treat ITO, tuning the energy‐level alignment of ITO‐perovskite layer to enhance hole extraction. Performance of such HTM‐free architecture (ITO/MAPbI_3_/C_60_/BCP/Ag) was then compared with analogous device configuration possessing HTM NiO*_x_* (e.g. ITO/NiO*_x_*/MAPbI_3_/C_60_/BCP/Ag), which revealed that devices with HTM NiO*_x_* exhibited a worse PCE and stability than as‐fabricated HTM‐free perovskite solar cells, thus highlighting such HTM‐free strategy for future low cost and simplified devices.^102^

Apart from well‐studied HTM‐free devices, ETM‐free perovskite solar cell is also explored and manifests high performance as well. Here, Liu et al. fabricated the device based on Ag/HTM/MAPbI_3_/ITO configuration and studied the device performance with or without ETM (ZnO). They demonstrated that the absence of ZnO ETM had slight impact on photovoltaic parameters of corresponding ETM‐free perovskite solar cell, which was mostly ascribed to the largely reduced charge recombination offsetting increased contact resistance at MAPbI_3_/ITO surface, resulting in a competitive PCE of 13.5% in regard to device with ETM. Moreover, as‐fabricated ETM‐free device possessed an enhanced thermal stability.^103^ These unique properties of HTM‐free and ETM‐free architectures consequently help form cost‐effective, simplified and high performance perovskite solar cells.

HTM and ETM‐free devices are particular architectures of perovskite solar cells with simplified configuration and reduced cost. However, high performance perovskite solar cells generally possess both HTM and ETM. As HTM‐free devices are investigated widely and have obtained reasonable PCE, future study should focus on ETM‐free configuration. Meanwhile, other approaches for controllable morphology and crystallization of perovskite layer, which improve photovoltaic performance of prospective HTM or ETM‐free devices, should be considered.

## 6 Conclusions

The current paper reviews strategies for enhancing performance of perovskite solar cells. Considering various device architectures, perovskite light harvesters, HTMs and ETMs are discussed separately in detail. Approaches for controllable morphology of perovskite layer are described such as thermal annealing, gas‐assisted technique, composition engineering and so forth. It is demonstrated that these processes are useful to realize more uniform perovskite layer with larger grain sized and better surface coverage, which strongly affect consequent photovoltaic performance of devices. For solvent and precursor engineering, appropriate additives are also expected to change the morphology and crystallization during perovskite materials preparation, which effectively produce high quality perovskite light absorber for superior devices.

Organic HTMs are being widely explored and focused mainly on additives and molecular engineering. Based on proper HOMO regarding perovskite layer, dopant‐free, cost‐effective, facile prepared, highly stable and additional light absorbed organic HTMs are favorable for achieving high performance perovskite solar cells. As for inorganic HTMs with intrinsic low cost and stable characteristics, optimization of interface and optoelectronic properties and thus enhancement of solar cell performance are the main focus.

Investigations of nanostructured ETMs are prevalent due to their outstanding electron transport pathway and charge extraction. TiO_2_ and ZnO nanostructures are commonly used as ETMs in perovskite solar cells, which are efficient but necessitate future improvement to form more suitable surface for perovskite absorber loading and optimize charge carriers dynamic. Novel potential ETMs and more nanostructures are also favorable. In addition, ETM modifications including n‐type doping and interface engineering are widely used for realizing high performance devices.

HTM and ETM‐free architectures, which enable fabrication of simple and low cost devices in comparison with the state‐of‐the‐art perovskite solar cells, are promising. However, further studies on enhancing photovoltaic parameters of corresponding perovskite solar cells are necessary.

To conclude, for future high performance perovskite solar cells, optimizations for morphology and crystallization of perovskite layer together with composition engineering are available strategies. Subsequent investigations should focus on achieving uniform, large‐scale and even innovative perovskite materials (Pb‐free) and corresponding low temperature techniques. While HTM and ETM‐free configurations are emerging, HTMs and ETMs for low cost and high performance devices are still essential to be explored to expand their family. Furthermore, long term stability, reproducibility and flexibility are all desirable for future commercialization of high performance perovskite solar cells.

## References

[1] a) M. Cheng , B. Xu , C. Chen , X. Yang , F. Zhang , Q. Tan , Y. Hua , L. Kloo , L. Sun , Adv. Energy Mater. 2015, 5, 1401720;

[2] a) W. Ni , X. Wan , M. Li , Y. Wang , Y. Chen , Chem. Commun. 2015, 51, 4936; 10.1039/c4cc09758k 25642992

[3] H. S. Jung , N. G. Park , Small 2015, 11, 10.25358818

[4] a) Q. Dong , Y. Fang , Y. Shao , P. Mulligan , J. Qiu , L. Cao , J. Huang , Science 2015, 347, 967; 25636799 10.1126/science.aaa5760

[5] A. Kojima , K. Teshima , Y. Shirai , T. Miyasaka , J. Am. Chem. Soc. 2009, 131, 6050. 19366264 10.1021/ja809598r

[6] H. S. Kim , C. R. Lee , J. H. Im , K. B. Lee , T. Moehl , A. Marchioro , S. J. Moon , R. Humphry‐Baker , J. H. Yum , J. E. Moser , M. Graetzel , N. G. Park , Sci. Rep. 2012, 2, 591. 22912919 10.1038/srep00591 PMC3423636

[7] J. Burschka , N. Pellet , S. J. Moon , R. Humphry Baker , P. Gao , M. K. Nazeeruddin , M. Graetzel , Nature 2013, 499, 316. 23842493 10.1038/nature12340

[8] N. J. Jeon , J. H. Noh , Y. C. Kim , W. S. Yang , S. Ryu , S. Il Seol , Nat. Mater. 2014, 13, 897. 24997740 10.1038/nmat4014

[9] W. S. Yang , J. H. Noh , N. J. Jeon , Y. C. Kim , S. Ryu , J. Seo , S. I. Seok , Science 2015, 348, 1234. 25999372 10.1126/science.aaa9272

[10] NREL, Best Research‐Cell efficiencies http://www.nrel.gov/ncpv/images/efficiency_chart.jpg, accessed: August, 2015.

[11] M. A. Green , A. Ho‐Baillie , H. J. Snaith , Nat. Photonics 2014, 8, 506.

[12] Y. Xu , L. Zhu , J. Shi , S. Lv , X. Xu , J. Xiao , J. Dong , H. Wu , Y. Luo , D. Li , Q. Meng , ACS Appl. Mater. Interfaces 2015, 7, 2242. 25587643 10.1021/am5057807

[13] a) X. P. Cui , K. J. Jiang , J. H. Huang , X. Q. Zhou , M. J. Su , S. G. Li , Q. Q. Zhang , L. M. Yang , Y. L. Song , Chem. Commun. 2015, 51, 1457; 10.1039/c4cc08269a 25493293

[14] L. Liu , A. Mei , T. Liu , P. Jiang , Y. Sheng , L. Zhang , H. Han , J. Am. Chem. Soc. 2015, 137, 1790. 25594109 10.1021/ja5125594

[15] N. G. Park , Mater. Today 2015, 18, 65.

[16] a) Y. Zhao , K. Zhu , J. Phys. Chem. C 2014, 118, 9412;

[17] Z. Wu , S. Bai , J. Xiang , Z. Yuan , Y. Yang , W. Cui , X. Gao , Z. Liu , Y. Jin , B. Sun , Nanoscale 2014, 6, 10505. 25081348 10.1039/c4nr03181d

[18] G. E. Eperon , V. M. Burlakov , P. Docampo , A. Goriely , H. J. Snaith , Adv. Funct. Mater. 2014, 24, 151.

[19] M. Xiao , F. Huang , W. Huang , Y. Dkhissi , Y. Zhu , J. Etheridge , A. Gray Weale , U. Bach , Y. B. Cheng , L. Spiccia , Angew. Chem. Int. Ed. 2014, 53, 9898. 10.1002/anie.201405334 25047967

[20] Y. Zhao , K. Zhu , J. Am. Chem. Soc. 2014, 136, 12241. 25118565 10.1021/ja5071398

[21] F. X. Xie , D. Zhang , H. Su , X. Ren , K. S. Wong , M. Graetzel , W. C. H. Choy , ACS Nano 2015, 9, 639. 25549113 10.1021/nn505978r

[22] S. Pathak , A. Sepe , A. Sadhanala , F. Deschler , A. Haghighirad , N. Sakai , K. C. Goedel , S. D. Stranks , N. Noel , M. Price , S. Huttner , N. A. Hawkins , R. H. Friend , U. Steiner , H. J. Snaith , ACS Nano 2015, 9, 2311. 25712705 10.1021/nn506465n

[23] Z. Xiao , C. Bi , Y. Shao , Q. Dong , Q. Wang , Y. Yuan , C. Wang , Y. Gao , J. Huang , Energy Environ. Sci. 2014, 7, 2619.

[24] Z. Xiao , Q. Dong , C. Bi , Y. Shao , Y. Yuan , J. Huang , Adv. Mater. 2014, 26, 6503. 25158905 10.1002/adma.201401685

[25] Q. Chen , H. Zhou , Z. Hong , S. Luo , H. S. Duan , H. H. Wang , Y. Liu , G. Li , Y. Yang , J. Am. Chem. Soc. 2014, 136, 622. 24359486 10.1021/ja411509g

[26] F. Huang , Y. Dkhissi , W. Huang , M. Xiao , I. Benesperi , S. Rubanov , Y. Zhu , X. Lin , L. Jiang , Y. Zhou , A. Gray Weale , J. Etheridge , C. R. McNeill , R. A. Caruso , U. Bach , L. Spiccia , Y. B. Cheng , Nano Energy 2014, 10, 10.

[27] W. Zhu , T. Yu , F. Li , C. Bao , H. Gao , Y. Yi , J. Yang , G. Fu , X. Zhou , Z. Zou , Nanoscale 2015, 7, 5427. 25733191 10.1039/c5nr00225g

[28] T. Du , N. Wang , H. Chen , H. Lin , H. He , ACS Appl. Mater. Interfaces 2015, 7, 3382. 25590573 10.1021/am508495r

[29] M. Liu , M. B. Johnston , H. J. Snaith , Nature 2013, 501, 395. 24025775 10.1038/nature12509

[30] P. Luo , Z. Liu , W. Xia , C. Yuan , J. Cheng , Y. Lu , ACS Appl. Mater. Interfaces 2015, 7, 2708. 25581720 10.1021/am5077588

[31] J. H. Im , I. H. Jang , N. Pellet , M. Graetzel , N. G. Park , Nat. Nanotechnol. 2014, 9, 927. 25173829 10.1038/nnano.2014.181

[32] F. Hao , C. C. Stoumpos , Z. Liu , R. P. H. Chang , M. G. Kanatzidis , J. Am. Chem. Soc. 2014, 136, 16411. 25374278 10.1021/ja509245x

[33] Y. Wu , A. Islam , X. Yang , C. Qin , J. Liu , K. Zhang , W. Peng , L. Han , Energy Environ. Sci. 2014, 7, 2934.

[34] W. Nie , H. Tsai , R. Asadpour , J. C. Blancon , A. J. Neukirch , G. Gupta , J. J. Crochet , M. Chhowalla , S. Tretiak , M. A. Alam , H.‐L. Wang , A. D. Mohite , Science 2015, 347, 522. 25635093 10.1126/science.aaa0472

[35] W. Zhang , M. Saliba , D. T. Moore , S. K. Pathak , M. T. Hoerantner , T. Stergiopoulos , S. D. Stranks , G. E. Eperon , J. A. Alexander‐Webber , A. Abate , A. Sadhanala , S. Yao , Y. Chen , R. H. Friend , L. A. Estroff , U. Wiesner , H. J. Snaith , Nat. Commun. 2015, 6, 6142. 25635571 10.1038/ncomms7142

[36] N. Pellet , P. Gao , G. Gregori , T. Y. Yang , M. K. Nazeeruddin , J. Maier , M. Graetzel , Angew. Chem. Int. Ed. 2014, 53, 3151. 10.1002/anie.201309361 24554633

[37] N. J. Jeon , J. H. Noh , W. S. Yang , Y. C. Kim , S. Ryu , J. Seo , S. I. Seok , Nature 2015, 517, 476. 25561177 10.1038/nature14133

[38] F. Wang , H. Yu , H. Xu , N. Zhao , Adv. Funct. Mater. 2015, 25, 1120.

[39] C. Y. Chang , C. Y. Chu , Y. C. Huang , C. W. Huang , S. Y. Chang , C. A. Chen , C. Y. Chao , W. F. Su , ACS Appl. Mater. Interfaces 2015, 7, 4955. 25679316 10.1021/acsami.5b00052

[40] Z. Zhu , Y. Bai , H. K. H. Lee , C. Mu , T. Zhang , L. Zhang , J. Wang , H. Yan , S. K. So , S. Yang , Adv. Funct. Mater. 2014, 24, 7357.

[41] a) D. Zhao , M. Sexton , H. Y. Park , G. Baure , J. C. Nino , F. So , Adv. Energy Mater. 2015, 5, 1401855;

[42] T. Leijtens , J. Lim , J. Teuscher , T. Park , H. J. Snaith , Adv. Mater. 2013, 25, 3227. 23637046 10.1002/adma.201300947

[43] J. Burschka , A. Dualeh , F. Kessler , E. Baranoff , N. L. Cevey Ha , C. Yi , M. K. Nazeeruddin , M. Graetzel , J. Am. Chem. Soc. 2011, 133, 18042. 21972850 10.1021/ja207367t

[44] a) D. Y. Chen , W. H. Tseng , S. P. Liang , C. I. Wu , C. W. Hsu , Y. Chi , W. Y. Hung , P. T. Chou , Phys. Chem. Chem. Phys. 2012, 14, 11689; 22824805 10.1039/c2cp41855j

[45] W. H. Nguyen , C. D. Bailie , E. L. Unger , M. D. McGehee , J. Am. Chem. Soc. 2014, 136, 10996. 25051503 10.1021/ja504539w

[46] W. Zhang , R. Zhu , F. Li , Q. Wang , B. Liu , J. Phys. Chem. C 2011, 115, 7038.

[47] F. Di Giacomo , S. Razza , F. Matteocci , A. D'Epifanio , S. Licoccia , T. M. Brown , A. Di Carlo , J. Power Sources 2014, 251, 152.

[48] Y. Zhang , W. Liu , F. Tan , Y. Gu , J. Power Sources 2015, 274, 1224.

[49] J. Liu , Y. Wu , C. Qin , X. Yang , T. Yasuda , A. Islam , K. Zhang , W. Peng , W. Chen , L. Han , Energy Environ. Sci. 2014, 7, 2963.

[50] P. Qin , S. Paek , M. I. Dar , N. Pellet , J. Ko , M. Graetzel , M. K. Nazeeruddin , J. Am. Chem. Soc. 2014, 136, 8516. 24866942 10.1021/ja503272q

[51] P. Qin , N. Tetreault , M. I. Dar , P. Gao , K. L. McCall , S. R. Rutter , S. D. Ogier , N. D. Forrest , J. S. Bissett , M. J. Simms , A. J. Page , R. Fisher , M. Graetzel , M. K. Nazeeruddin , Adv. Energy Mater. 2015, 5, 1400980.

[52] a) A. Tomkeviciene , J. V. Grazulevicius , K. Kazlauskas , A. Gruodis , S. Jursenas , T. H. Ke , C. C. Wu , J. Phys. Chem. C 2011, 115, 4887;

[53] B. Xu , E. Sheibani , P. Liu , J. Zhang , H. Tian , N. Vlachopoulos , G. Boschloo , L. Kloo , A. Hagfeldt , L. Sun , Adv. Mater. 2014, 26, 6629. 25124337 10.1002/adma.201402415

[54] N. J. Jeon , J. Lee , J. H. Noh , M. K. Nazeeruddin , M. Graetzel , S. I. Seok , J. Am. Chem. Soc. 2013, 135, 19087. 24313292 10.1021/ja410659k

[55] H. Choi , S. Paek , N. Lim , Y. H. Lee , M. K. Nazeeruddin , J. Ko , Chem.‐Eur. J. 2014, 20, 10894. 25100664 10.1002/chem.201403807

[56] J. Seo , S. Park , Y. C. Kim , N. J. Jeon , J. H. Noh , S. C. Yoon , S. I. Seok , Energy Environ. Sci. 2014, 7, 2642.

[57] a) J. H. Heo , S. H. Im , J. H. Noh , T. N. Mandal , C. S. Lim , J. A. Chang , Y. H. Lee , H. j. Kim , A. Sarkar , M. K. Nazeeruddin , M. Graetzel , S. I. Seok , Nat. Photonics 2013, 7, 487;

[58] S. Ryu , J. H. Noh , N. J. Jeon , Y. C. Kim , S. Yang , J. Seo , S. I. Seok , Energy Environ. Sci. 2014, 7, 2614.

[59] J. A. Christians , R. C. M. Fung , P. V. Kamat , J. Am. Chem. Soc. 2014, 136, 758. 24350620 10.1021/ja411014k

[60] M. D. Irwin , B. Buchholz , A. W. Hains , R. P. H. Chang , T. J. Marks , Proc.e Natl.Acad. Sci. USA 2008, 105, 2783.

[61] A. S. Subbiah , A. Halder , S. Ghosh , N. Mahuli , G. Hodes , S. K. Sarkar , J. Phys. Chem. Lett. 2014, 5, 1748. 26270378 10.1021/jz500645n

[62] Z. Zhu , Y. Bai , T. Zhang , Z. Liu , X. Long , Z. Wei , Z. Wang , L. Zhang , J. Wang , F. Yan , S. Yang , Angew. Chem. Int. Ed. 2014, 53, 12571. 10.1002/anie.201405176 25044246

[63] K. C. Wang , J. Y. Jeng , P. S. Shen , Y. C. Chang , E. W. G. Diau , C. H. Tsai , T. Y. Chao , H. C. Hsu , P. Y. Lin , P. Chen , T. F. Guo , T. C. Wen , Sci. Rep. 2014, 4, 4756. 24755642 10.1038/srep04756 PMC3996464

[64] J. H. Kim , P. W. Liang , S. T. Williams , N. Cho , C. C. Chueh , M. S. Glaz , D. S. Ginger , A. K. Y. Jen , Adv. Mater. 2015, 27, 695. 25449020 10.1002/adma.201404189

[65] P. Qin , S. Tanaka , S. Ito , N. Tetreault , K. Manabe , H. Nishino , M. K. Nazeeruddin , M. Graetzel , Nat. Commun. 2014, 5, 3834. 24815001 10.1038/ncomms4834

[66] a) C. Xu , J. Wu , U. V. Desai , D. Gao , J. Am. Chem. Soc. 2011, 133, 8122; 21526854 10.1021/ja202135n

[67] Q. Huang , G. Zhou , L. Fang , L. Hu , Z.‐S. Wang , Energy Environ. Sci. 2011, 4, 2145.

[68] J. Qiu , Y. Qiu , K. Yan , M. Zhong , C. Mu , H. Yan , S. Yang , Nanoscale 2013, 5, 3245. 23508213 10.1039/c3nr00218g

[69] X. Wang , Z. Li , W. Xu , S. A. Kulkarni , S. K. Batabyal , S. Zhang , A. Cao , L. H. Wong , Nano Energy 2015, 11, 728.

[70] S. H. Kang , S. H. Choi , M. S. Kang , J. Y. Kim , H. S. Kim , T. Hyeon , Y. E. Sung , Adv. Mater. 2008, 20, 54.

[71] H. S. Kim , J. W. Lee , N. Yantara , P. P. Boix , S. A. Kulkarni , S. Mhaisalkar , M. Graetzel , N. G. Park , Nano Lett. 2013, 13, 2412. 23672481 10.1021/nl400286w

[72] B. Cai , D. Zhong , Z. Yang , B. Huang , S. Miao , W. H. Zhang , J. Qiu , C. Li , J. Mater. Chem. C 2015, 3, 729.

[73] X. Xin , M. Scheiner , M. Ye , Z. Lin , Langmuir 2011, 27, 14594. 22013973 10.1021/la2034627

[74] A. Yella , L. P. Heiniger , P. Gao , M. K. Nazeeruddin , M. Graetzel , Nano Lett. 2014, 14, 2591. 24628563 10.1021/nl500399m

[75] J. T. W. Wang , J. M. Ball , E. M. Barea , A. Abate , J. A. Alexander Webber , J. Huang , M. Saliba , I. Mora Sero , J. Bisquert , H. J. Snaith , R. J. Nicholas , Nano Lett. 2014, 14, 724. 24341922 10.1021/nl403997a

[76] K. Wojciechowski , M. Saliba , T. Leijtens , A. Abate , H. J. Snaith , Energy Environ. Sci. 2014, 7, 1142.

[77] D. Zhong , B. Cai , X. Wang , Z. Yang , Y. Xing , S. Miao , W. H. Zhang , C. Li , Nano Energy 2015, 11, 409.

[78] M. H. Kumar , N. Yantara , S. Dharani , M. Graetzel , S. Mhaisalkar , P. P. Boix , N. Mathews , Chem. Commun. 2013, 49, 11089. 10.1039/c3cc46534a 24141601

[79] D. Y. Son , J. H. Im , H. S. Kim , N. G. Park , J. Phys. Chem. C 2014, 118, 16567.

[80] L. Liang , Z. Huang , L. Cai , W. Chen , B. Wang , K. Chen , H. Bai , Q. Tian , B. Fan , ACS Appl. Mater. Interfaces 2014, 6, 20585. 25405518 10.1021/am506672j

[81] D. Liu , T. L. Kelly , Nat. Photonics 2014, 8, 133.

[82] J. Zhang , P. Barboux , T. Pauporte , Adv. Energy Mater. 2014, 4, 1400932.

[83] K. Mahmood , B. S. Swain , A. Amassian , Nanoscale 2014, 6, 14674. 25373624 10.1039/c4nr04383a

[84] Z. Zhu , J. Ma , Z. Wang , C. Mu , Z. Fan , L. Du , Y. Bai , L. Fan , H. Yan , D. L. Phillips , S. Yang , J. Am. Chem. Soc. 2014, 136, 3760. 24558950 10.1021/ja4132246

[85] H. Zhang , H. Azimi , Y. Hou , T. Ameri , T. Przybilla , E. Spiecker , M. Kraft , U. Scherf , C. J. Brabec , Chem. Mater. 2014, 26, 5190.

[86] H. Azimi , T. Ameri , H. Zhang , Y. Hou , C. O. R. Quiroz , J. Min , M. Hu , Z.‐G. Zhang , T. Przybilla , G. J. Matt , E. Spiecker , Y. Li , C. J. Brabec , Adv. Energy Mater. 2015, 5, 1401692.

[87] J. Min , Z.‐G. Zhang , Y. Hou , C. O. R. Quiroz , T. Przybilla , C. Bronnbauer , F. Guo , K. Forberich , H. Azimi , T. Ameri , E. Spiecker , Y. Li , C. J. Brabec , Chem. Mater. 2015, 27, 227.

[88] J. Deng , M. Wang , J. Liu , X. Song , Z. Yang , J. Colloid. Interface. Sci. 2014, 418, 277. 24461846 10.1016/j.jcis.2013.11.017

[89] J. Dong , Y. Zhao , J. Shi , H. Wei , J. Xiao , X. Xu , J. Luo , J. Xu , D. Li , Y. Luo , Q. Meng , Chem. Commun. 2014, 50, 13381. 10.1039/c4cc04908j 25233329

[90] K. Wojciechowski , S. D. Stranks , A. Abate , G. Sadoughi , A. Sadhanala , N. Kopidakis , G. Rumbles , C. Z. Li , R. H. Friend , A. K. Y. Jen , H. J. Snaith , ACS Nano 2014, 8, 12701. 25415931 10.1021/nn505723h

[91] L. Zuo , Z. Gu , T. Ye , W. Fu , G. Wu , H. Li , H. Chen , J. Am. Chem. Soc. 2015, 137, 2674. 25650811 10.1021/ja512518r

[92] C. Liu , K. Wang , P. Du , T. Meng , X. Yu , S. Z. D. Cheng , X. Gong , ACS Appl. Mater. Interfaces 2015, 7, 1153. 25513751 10.1021/am506869k

[93] H. Zhou , Q. Chen , G. Li , S. Luo , T. b. Song , H. S. Duan , Z. Hong , J. You , Y. Liu , Y. Yang , Science 2014, 345, 542. 25082698 10.1126/science.1254050

[94] V. D'Innocenzo , G. Grancini , M. J. P. Alcocer , A. R. S. Kandada , S. D. Stranks , M. M. Lee , G. Lanzani , H. J. Snaith , A. Petrozza , Nat. Commun. 2014, 5, 3586. 24710005 10.1038/ncomms4586

[95] L. Etgar , P. Gao , Z. Xue , Q. Peng , A. K. Chandiran , B. Liu , M. K. Nazeeruddin , M. Graetzel , J. Am. Chem. Soc. 2012, 134, 17396. 23043296 10.1021/ja307789s

[96] W. Abu Laban , L. Etgar , Energy Environ. Sci. 2013, 6, 3249.

[97] S. Aharon , S. Gamliel , B. El Cohen , L. Etgar , Phys. Chem. Chem. Phys. 2014, 16, 10512. 24736900 10.1039/c4cp00460d

[98] J. Shi , J. Dong , S. Lv , Y. Xu , L. Zhu , J. Xiao , X. Xu , H. Wu , D. Li , Y. Luo , Q. Meng , Appl. Phys. Lett. 2014, 104.

[99] J. Shi , Y. Luo , H. Wei , J. Luo , J. Dong , S. Lv , J. Xiao , Y. Xu , L. Zhu , X. Xu , H. Wu , D. Li , Q. Meng , ACS Appl. Mater. Interfaces 2014, 6, 9711. 24830329 10.1021/am502131t

[100] H. Wei , J. Shi , X. Xu , J. Xiao , J. Luo , J. Dong , S. Lv , L. Zhu , H. Wu , D. Li , Y. Luo , Q. Meng , Q. Chen , Phys. Chem. Chem. Phys. 2015, 17, 4937. 25594083 10.1039/c4cp04902k

[101] A. Mei , X. Li , L. Liu , Z. Ku , T. Liu , Y. Rong , M. Xu , M. Hu , J. Chen , Y. Yang , M. Graetzel , H. Han , Science 2014, 345, 295. 25035487 10.1126/science.1254763

[102] Y. Li , S. Ye , W. Sun , W. Yan , Y. Li , Z. Bian , Z. Liu , S. Wang , C. Huang , J. Mater. Chem. A, DOI:10.1039/c5ta05989e.

[103] D. Liu , J. Yang , T. L. Kelly , J. Am. Chem. Soc. 2014, 136, 17116. 25405271 10.1021/ja508758k

